# Antioxidant Activity and DAF-16-Associated Lifespan-Promoting Effects of Essential Oil from *Melastoma sanguineum* Sims in *Caenorhabditis elegans*

**DOI:** 10.3390/molecules31203585

**Published:** 2026-10-09

**Authors:** Xue Cui, Xu Liu, Jing-Shan Wu, Ying Han, Heng-Jia Wei, Ying-Xiu Li

**Affiliations:** Marine College, Shandong University, Weihai 264209, China; 202437781@mail.sdu.edu.cn (X.C.); deanwhite@sdu.edu.cn (X.L.); 202538038@mail.sdu.edu.cn (Y.H.); 202538043@mail.sdu.edu.cn (H.-J.W.)

**Keywords:** *Melastoma sanguineum*, essential oil, *Caenorhabditis elegans*, longevity, oxidative stress, DAF-16

## Abstract

*Melastoma sanguineum* Sims is a traditional medicinal plant with edible fruits, but little is known about the biological activities of its essential oil. In this study, *Caenorhabditis elegans* was used as an animal model to investigate the antioxidant and lifespan-promoting effects of *M. sanguineum* essential oil, designated EO-2141. EO-2141 showed in vitro antioxidant activity. In *C. elegans*, EO-2141 significantly extended mean lifespan and reduced lipofuscin accumulation. It also enhanced resistance to oxidative and thermal stress and reduced DCF fluorescence. In addition, EO-2141 reduced MDA levels and increased SOD and GSH-PX activities. Gene-expression analysis showed that EO-2141 upregulated *daf-16* and the stress-responsive genes *sod-3*, *ctl-2*, and *hsp-16.2*, while reducing *akt-1* expression. DAF-16::GFP analysis further showed that EO-2141 increased the proportion of worms exhibiting nuclear DAF-16 localization. Moreover, EO-2141 failed to produce a significant lifespan extension in *daf-16(mgDf50)* mutants, further implicating DAF-16 in the longevity response to EO-2141. These findings demonstrate the antioxidant and lifespan-promoting effects of EO-2141 in *C. elegans* and provide transcriptional, subcellular-localization, and genetic evidence supporting the involvement of DAF-16 in its longevity response.

## 1. Introduction

With the rapid aging of the global population, aging-related diseases have become major public health challenges [1]. Oxidative stress is an important contributor to aging and aging-related functional decline, as excessive reactive oxygen species (ROS) can damage proteins, lipids, and DNA [2,3]. Plant-derived natural products contain diverse secondary metabolites and may exert antioxidant effects through direct free-radical scavenging, enhancement of endogenous antioxidant defenses, and modulation of cellular stress-response pathways [4,5]. They have therefore attracted increasing attention for their potential antioxidant and health-promoting effects.

*Melastoma sanguineum* Sims is a traditional medicinal plant widely distributed in southern China, and its ripe fruits are edible [6]. *Melastoma* species contain diverse bioactive constituents, including flavonoids, phenolics, terpenoids, and steroids, and their extracts have shown antioxidant, anti-inflammatory, antimicrobial, and other biological activities [7]. Despite these studies on the phytochemical constituents and biological activities of *Melastoma* species, much less is known about their essential oils. In particular, the biological activities of *M. sanguineum* essential oil remain poorly characterized, especially its effects on oxidative stress and aging-related phenotypes.

*Caenorhabditis elegans* is widely used as an in vivo model for studying aging, oxidative stress, and the biological activities of natural products [8]. Its short lifespan, transparent body, and genetic tractability facilitate aging-related studies [9]. Age-associated phenotypes, including lipofuscin accumulation and changes in stress resistance, provide useful readouts for evaluating the effects of natural products on aging [10]. At the molecular level, the insulin/insulin-like signaling (IIS) pathway is an important regulator of lifespan and stress responses in *C. elegans* [11]. Signaling through the DAF-2 insulin/IGF-1 receptor and downstream AKT kinases regulates the activity and subcellular localization of DAF-16, a FOXO transcription factor that plays a key role in lifespan and stress resistance. Nuclear DAF-16 regulates genes involved in stress adaptation and longevity, including *sod-3*, *ctl-2*, and *hsp-16.2* [12]. Oxidative stress responses are also regulated by SKN-1, an ortholog of mammalian Nrf transcription factors involved in redox homeostasis [13]. Several plant-derived natural products, including essential oils, have been reported to improve stress resistance and longevity in *C. elegans*, with DAF-16 implicated in some of these effects [14,15].

Based on this background, the present study obtained an essential oil from *M. sanguineum*, designated EO-2141, and characterized its chemical composition and in vitro antioxidant activity. Using *C. elegans* as an in vivo model, we evaluated the effects of EO-2141 on lifespan, aging-related phenotypes, stress resistance, and oxidative status. We further examined antioxidant enzyme activities and stress-related gene expression and assessed the involvement of DAF-16 through DAF-16::GFP nuclear localization and lifespan analysis in *daf-16(mgDf50)* mutant worms. To the best of our knowledge, this is the first study to investigate the antioxidant and lifespan-promoting effects of *M. sanguineum* essential oil in *C. elegans* and to examine the involvement of DAF-16 in its longevity response.

## 2. Results

### 2.1. Chemical Composition of Melastoma sanguineum Essential Oil (EO-2141)

EO-2141 was obtained as a pale-yellow essential oil by hydrodistillation of fresh leaves and stems of *M. sanguineum*, with a yield of 0.15 ± 0.01 mL/kg fresh weight. The chemical composition of EO-2141 was characterized by GC-FID and GC-MS. A total of 91 compounds were identified, accounting for 98.99% of the total GC peak area. The relative abundance of each constituent was determined by GC-FID peak-area normalization. As shown in Table S1, the major constituents were (E)-2-hexenal (21.31%), *n*-hexadecanoic acid (16.40%), ethyl linolenate (3.61%), mandenol (3.56%), and squalene (3.19%).

### 2.2. In Vitro Antioxidant Activity of EO-2141

The antioxidant activity of EO-2141 was evaluated using DPPH, ABTS, and ferric reducing antioxidant power (FRAP) assays [16], and the results are summarized in Table 1. In the DPPH assay, EO-2141 exhibited relatively weak radical-scavenging activity, with an IC_50_ value above 10 mg/mL. At the highest concentration tested (10 mg/mL), the DPPH radical-scavenging rate was 23.80%, whereas the IC_50_ values of the positive controls BHT and Trolox were 0.043 ± 0.0012 mg/mL and 0.012 ± 0.0008 mg/mL, respectively. In the ABTS assay, EO-2141 showed greater radical-scavenging activity, with an IC_50_ value of 3.72 ± 0.232 mg/mL, although its activity was lower than that of BHT (0.015 ± 0.0003 mg/mL) and Trolox (0.010 ± 0.0005 mg/mL). The FRAP value of EO-2141 was 62.88 ± 2.75 μmol TE/g.

### 2.3. EO-2141 Extends the Lifespan of C. elegans

**Figure 1:**
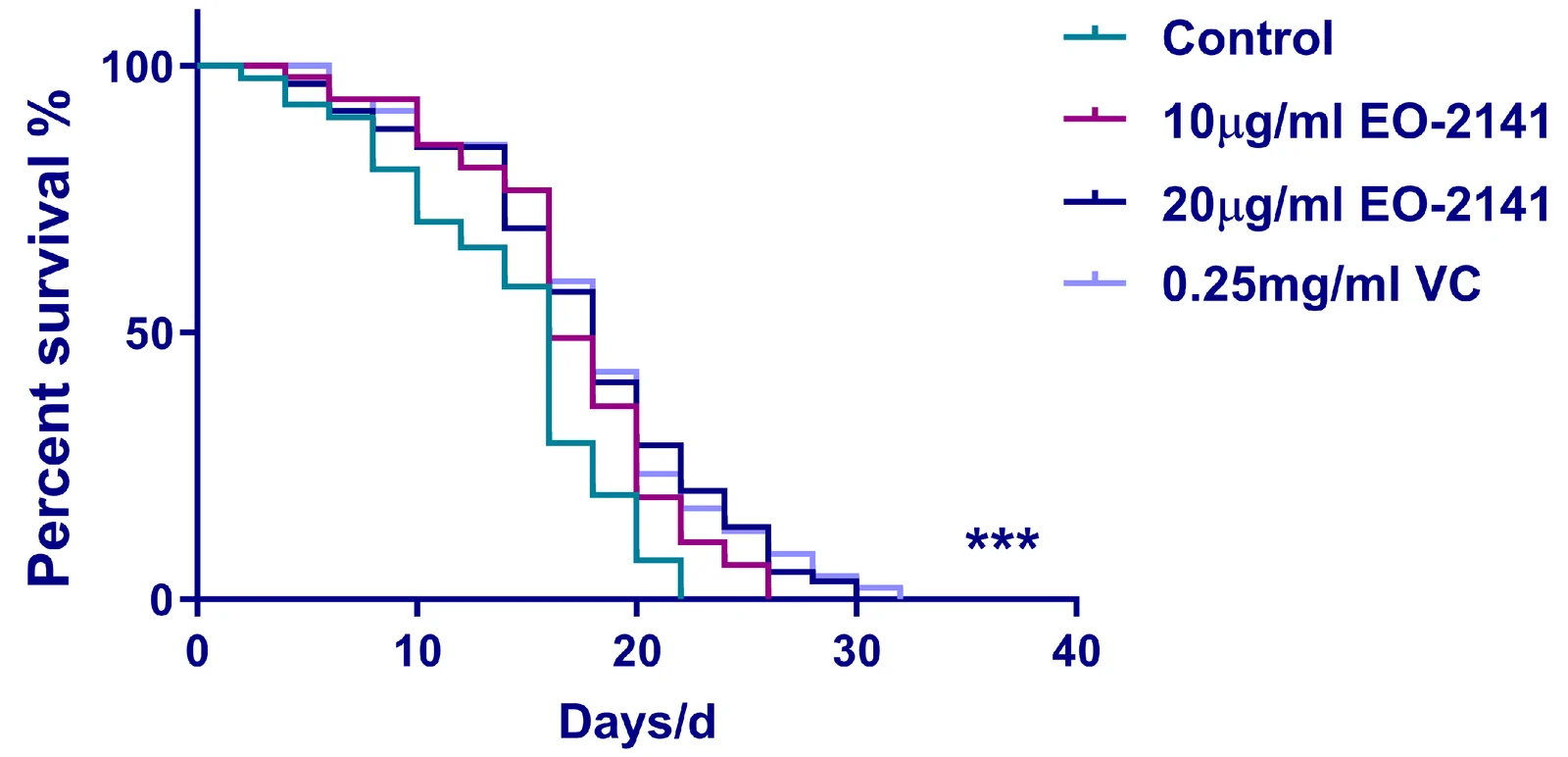
Effect of EO-2141 on the lifespan of wild-type *C. elegans* (N2). Survival curves of wild-type N2 worms under different treatment conditions. The control group was maintained on NGM plates containing vehicle only (0.1% ethanol), while the treatment groups received EO-2141 at concentrations of 10 or 20 μg/mL. Vitamin C (VC, 0.25 mg/mL) was used as the positive control. Statistical significance was determined using the log-rank test (*** *p* < 0.001).

Physiological traits such as locomotor activity, pharyngeal pumping, and reproductive performance change with age in *C. elegans* and are commonly used to assess age-related physiological function [17]. To determine whether EO-2141 affected general physiological functions in *C. elegans*, locomotor activity, pharyngeal pumping, fecundity, and body size were examined before lifespan analysis. Two concentrations of EO-2141 (10 and 20 μg/mL) were evaluated. As shown in Supplementary Figures S1–S4, EO-2141 treatment did not significantly affect locomotor activity, pharyngeal pumping rate, fecundity, or body size compared with the control group. EO-2141 also did not significantly affect food clearance (Figure S5), indicating that food consumption was not markedly altered under the experimental conditions. These results indicate that EO-2141 did not significantly alter the physiological and feeding parameters examined. The effect of EO-2141 on lifespan was subsequently evaluated in wild-type N2 worms. As shown in Figure 1 and Table 2, treatment with 10 and 20 μg/mL EO-2141 significantly increased mean lifespan by 16.22% and 26.33%, respectively, compared with the control group (10 μg/mL, *p* < 0.05; 20 μg/mL, *p* < 0.001). The positive control, vitamin C (0.25 mg/mL), increased mean lifespan by 29.42%. The median lifespan increased from 16 days in the control group to 18 days in worms treated with 20 μg/mL EO-2141. Thus, EO-2141 significantly extended lifespan in *C. elegans*, with a greater effect observed at 20 μg/mL. Therefore, 20 μg/mL EO-2141 was selected for subsequent analyses related to DAF-16 involvement.

### 2.4. EO-2141 Reduces Lipofuscin Accumulation in C. elegans

**Figure 2:**
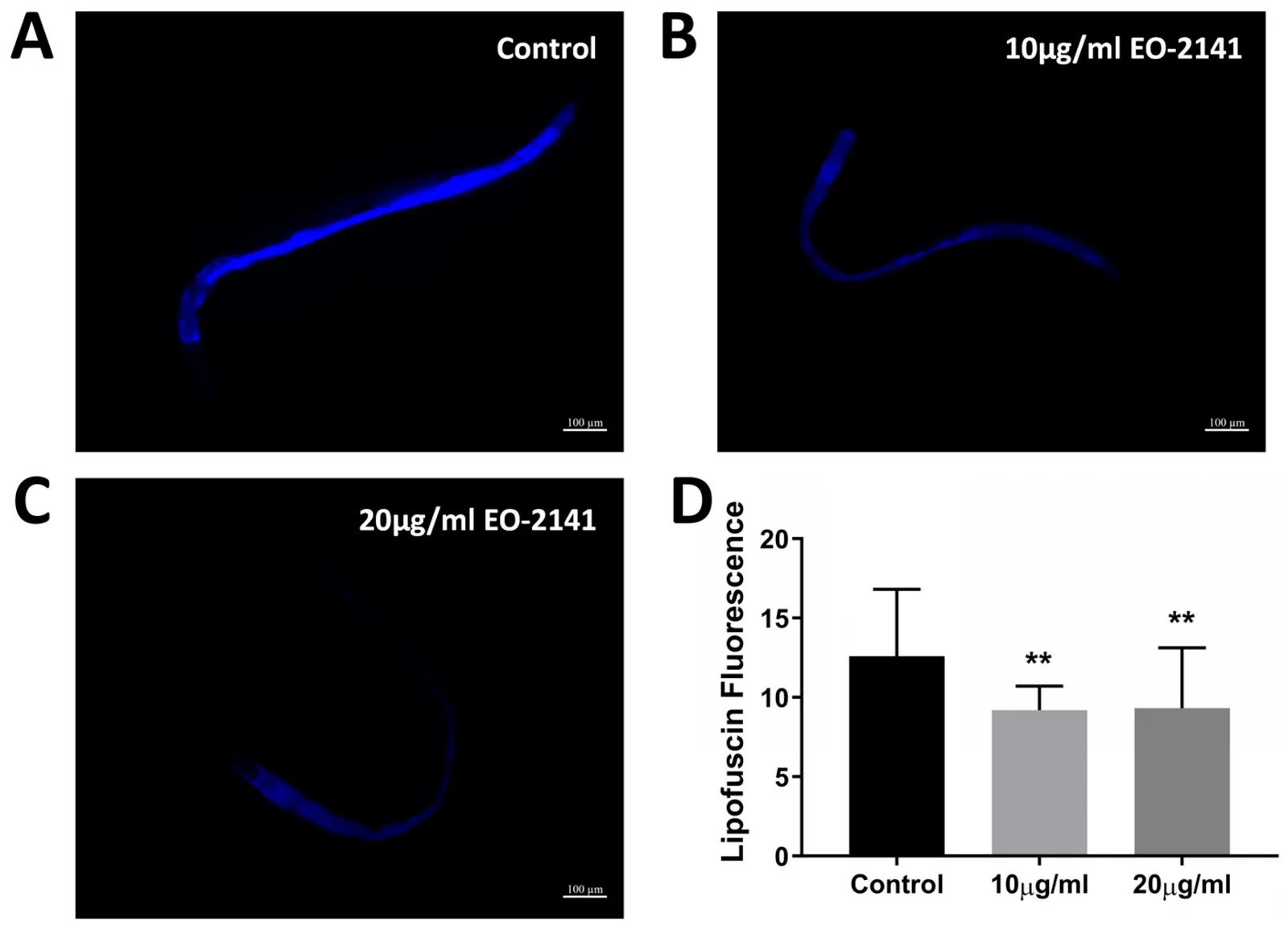
Effects of EO-2141 on lipofuscin accumulation in *C. elegans.* (**A**–**C**) Representative fluorescence images showing intestinal lipofuscin autofluorescence in worms from different treatment groups (excitation wavelength, 340 nm; emission wavelength, 430 nm). (**D**) Quantitative analysis of lipofuscin fluorescence intensity using ImageJ software. Data are presented as the mean ± SEM from three independent biological replicates, with 10 worms analyzed per treatment group in each replicate. Statistical significance was evaluated using one-way ANOVA followed by Dunnett’s post hoc test (** *p* < 0.01). Scale bars: 200 μm.

Lipofuscin is an age-associated autofluorescent pigment that accumulates in *C. elegans* during aging and has been widely used as an indicator of age-related changes [18,19]. To evaluate the effect of EO-2141 on lipofuscin accumulation, intestinal lipofuscin autofluorescence was measured using an inverted fluorescence microscope at excitation and emission wavelengths of 340 and 430 nm, respectively, and fluorescence intensity was quantified using ImageJ software (version 1.54d). As shown in Figure 2, EO-2141 treatment significantly reduced intestinal lipofuscin autofluorescence compared with the control group at both 10 μg/mL (*p* = 0.0026) and 20 μg/mL (*p* = 0.0085). Specifically, fluorescence intensity decreased by 26.98% and 26.04% in worms treated with 10 and 20 μg/mL EO-2141, respectively. These results indicate that EO-2141 reduces age-associated lipofuscin accumulation in *C. elegans*.

### 2.5. EO-2141 Enhances Resistance to Oxidative and Thermal Stress in C. elegans

**Figure 3:**
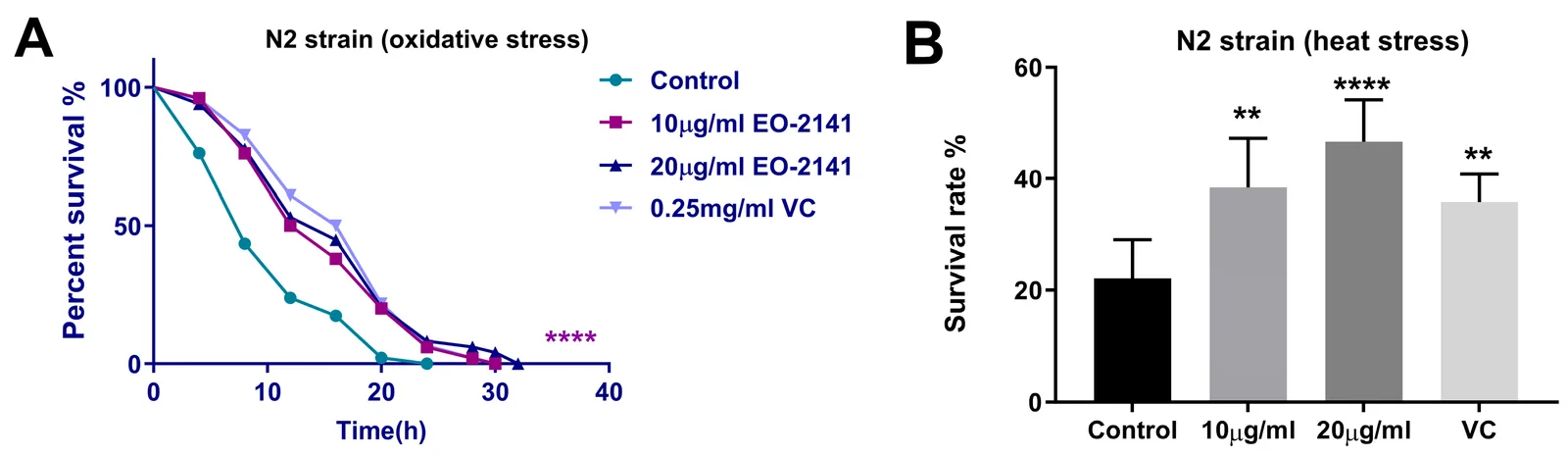
Effects of EO-2141 on stress resistance in *C. elegans*. (**A**) Effects of EO-2141 on oxidative stress resistance in *C. elegans* under paraquat-induced oxidative stress conditions (survival curves). (**B**) Effects of EO-2141 on thermotolerance in *C. elegans* following heat stress at 35 °C. Data are presented as the mean ± SEM. Statistical significance was determined using the log-rank test for panel (**A**) and one-way ANOVA for panel (**B**). ** *p* < 0.01 and **** *p* < 0.0001 versus the control group.

A decline in stress resistance is associated with aging, and the ability of *C. elegans* to tolerate environmental stress is closely related to longevity [20]. To investigate the effects of EO-2141 on stress resistance, paraquat-induced oxidative stress and heat-stress assays were performed. In the acute oxidative stress assay, worms were exposed to paraquat following EO-2141 treatment. As shown in Figure 3A, worms treated with either concentration of EO-2141, as well as those in the positive control group (vitamin C, VC), showed significantly prolonged survival compared with the control group (log-rank test, *p* < 0.001). The mean survival times of worms treated with 10 and 20 μg/mL EO-2141 were 1.75- and 2.00-fold those of the control group, respectively, while the corresponding maximum survival times were 1.50- and 1.60-fold those of the control group. The effect of EO-2141 on thermal stress resistance was further evaluated using a heat-shock assay. Following exposure to 35 °C for 5 h and recovery at 20 °C for 24 h, the survival rate of the control group was 22.13 ± 2.81%, compared with 38.42 ± 3.62%, 46.67 ± 3.07%, and 35.78 ± 2.05% in worms treated with 10 μg/mL EO-2141, 20 μg/mL EO-2141, and VC, respectively (Figure 3B). Compared with the control group, survival under heat stress was significantly increased by treatment with 10 μg/mL EO-2141 (*p* < 0.01), 20 μg/mL EO-2141 (*p* < 0.0001), and VC (*p* < 0.01). EO-2141 therefore enhanced resistance to both oxidative and thermal stress in *C. elegans*.

### 2.6. EO-2141 Reduces H_2_DCFH-DA-Derived DCF Fluorescence in C. elegans

**Figure 4:**
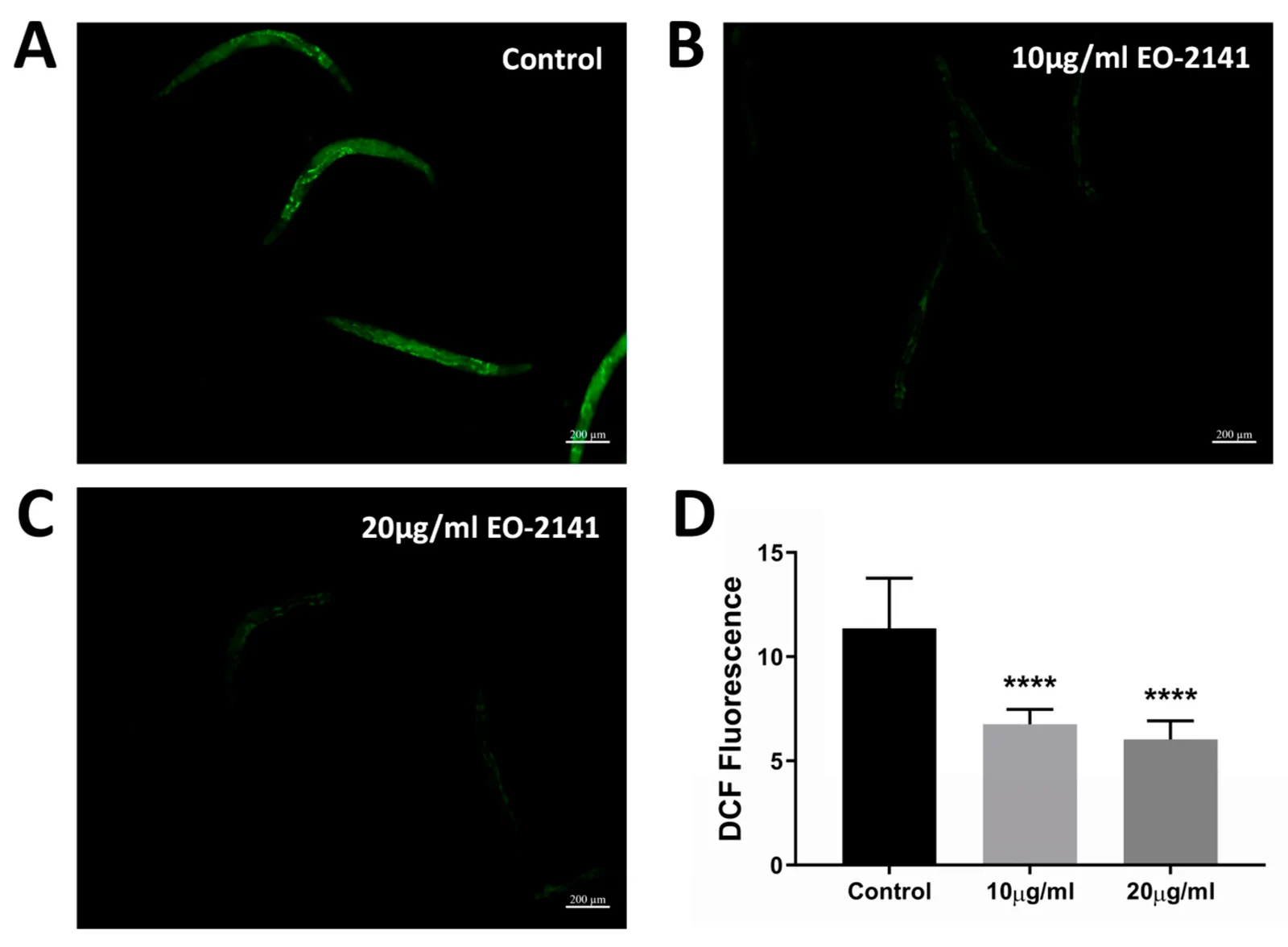
Effects of EO-2141 on H_2_DCFH-DA-derived DCF fluorescence in *C. elegans*. (**A**–**C**) Representative fluorescence images of H_2_DCFH-DA-stained worms from different treatment groups (5× magnification). (**D**) Quantitative analysis of DCF fluorescence intensity using ImageJ software. Data are presented as the mean ± SEM from three independent biological replicates, with 20 worms analyzed per treatment group in each replicate. Statistical significance was determined by one-way ANOVA followed by Dunnett’s post hoc test. **** *p* < 0.0001 versus the control group. Scale bar = 200 μm.

Excessive accumulation of reactive oxygen species (ROS) is considered a major contributor to oxidative damage and age-related physiological decline [21]. Because EO-2141 significantly enhanced oxidative stress resistance in *C. elegans*, its effect on intracellular oxidative status was further investigated using the fluorescent probe H_2_DCFH-DA. Representative fluorescence images are shown in Figure 4A–C. Compared with the control group, worms treated with 10 μg/mL and 20 μg/mL EO-2141 showed markedly weaker green fluorescence signals. Quantitative analysis using ImageJ software confirmed that the relative fluorescence intensity was significantly reduced by 39.70% and 46.84% in the 10 μg/mL and 20 μg/mL treatment groups, respectively (*p* < 0.0001; Figure 4D). EO-2141 therefore significantly reduced H_2_DCFH-DA-derived DCF fluorescence in *C. elegans*.

### 2.7. EO-2141 Modulates Antioxidant Enzyme Activities and MDA Levels in C. elegans

**Figure 5:**
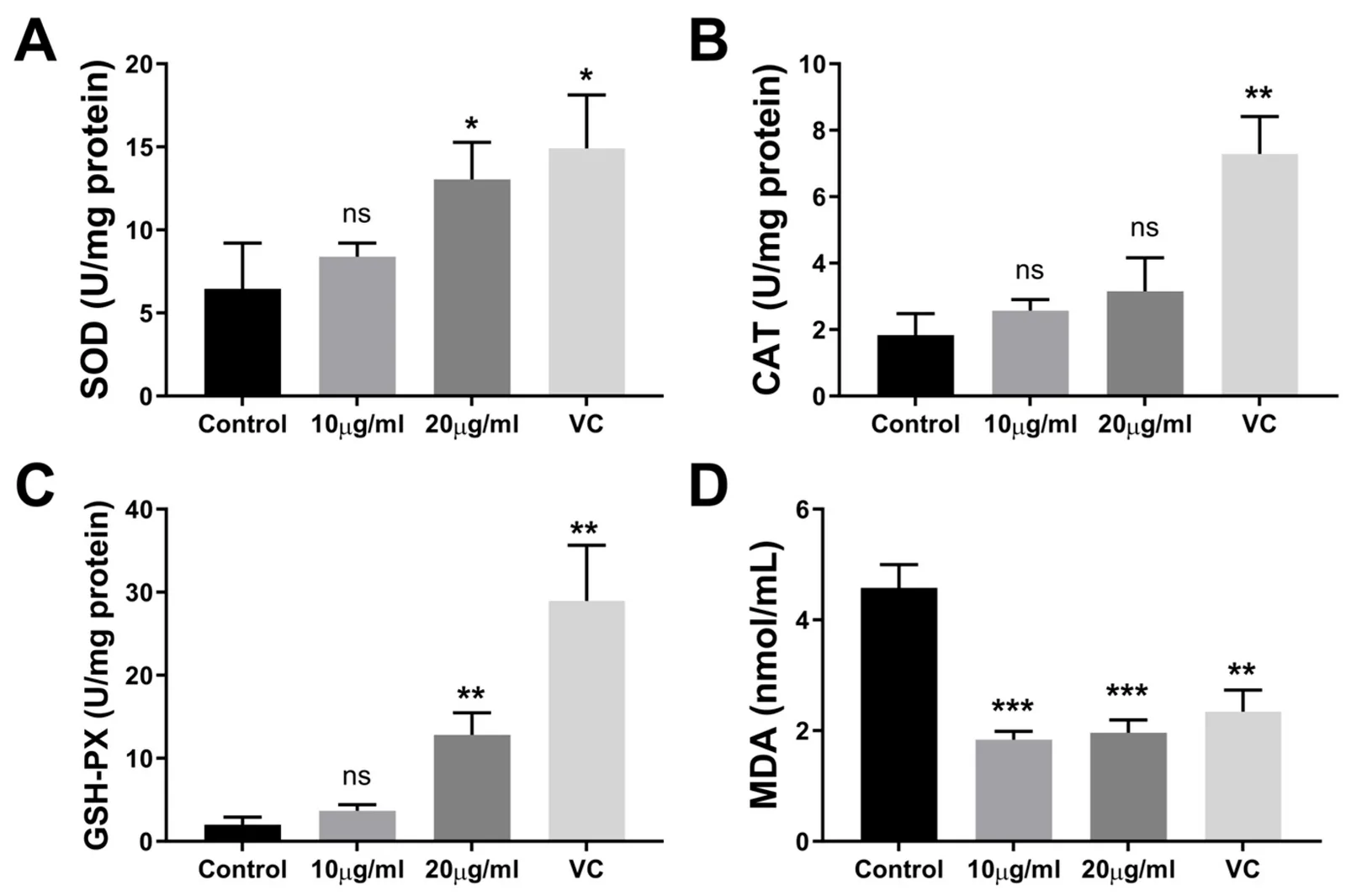
Effects of EO-2141 on antioxidant enzyme activities and MDA levels in *C. elegans*. (**A**) SOD activity. (**B**) CAT activity. (**C**) GSH-PX activity. (**D**) MDA content. Data are presented as the mean ± SEM. Statistical significance was determined by one-way ANOVA followed by Dunnett’s post hoc test. * *p* < 0.05, ** *p* < 0.01, *** *p* < 0.001, and ns, not significant (*p* > 0.05).

The cellular antioxidant defense system contributes to redox homeostasis through enzymatic and non-enzymatic mechanisms. Superoxide dismutase (SOD), catalase (CAT), and glutathione peroxidase (GSH-PX) are important antioxidant enzymes, whereas malondialdehyde (MDA), a product of lipid peroxidation, is commonly used as an indicator of oxidative damage [13]. To evaluate the effects of EO-2141 on antioxidant status, antioxidant enzyme activities and MDA levels were measured following EO-2141 treatment. As shown in Figure 5A–C, treatment with 20 μg/mL EO-2141 significantly increased SOD (*p* = 0.0327) and GSH-PX (*p* = 0.0026) activities compared with the control group, whereas CAT activity was not significantly altered. No significant changes in SOD, CAT, or GSH-PX activities were observed following treatment with 10 μg/mL EO-2141. MDA levels were significantly reduced in both EO-2141-treated groups (Figure 5D). Compared with the control group, MDA levels decreased by 59.82% and 58.21% following treatment with 10 and 20 μg/mL EO-2141, respectively (10 μg/mL, *p* = 0.0004; 20 μg/mL, *p* = 0.0007). The positive control, vitamin C (VC), significantly increased SOD (*p* = 0.0261), CAT (*p* = 0.0019), and GSH-PX (*p* = 0.0023) activities and reduced MDA levels (*p* = 0.0025). These results indicate that EO-2141 modulates antioxidant enzyme activity and reduces lipid peroxidation in *C. elegans*.

### 2.8. EO-2141 Modulates the Expression of IIS-Related and Antioxidant/Stress-Response Genes in C. elegans

**Figure 6:**
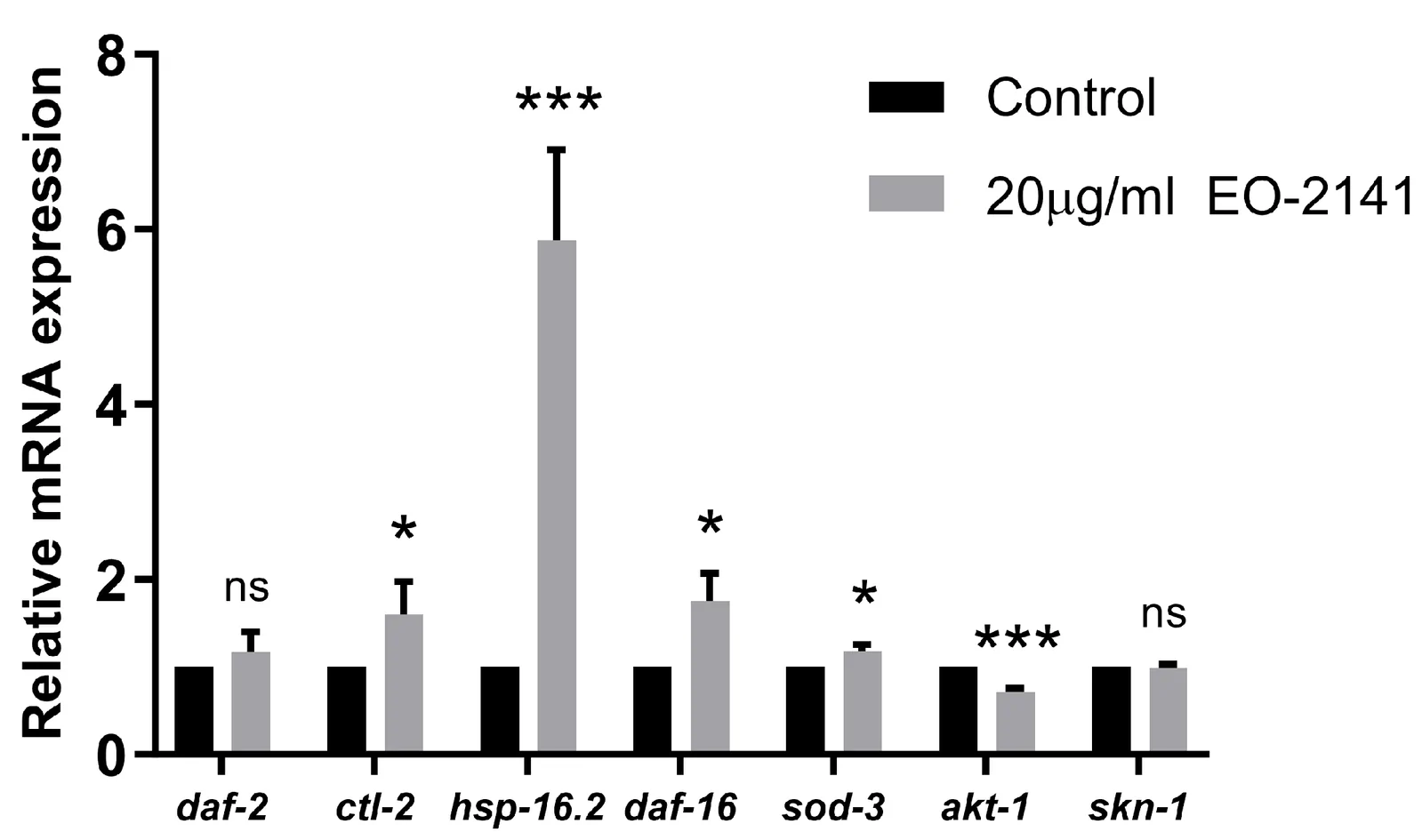
Effects of EO-2141 on the expression of antioxidant- and aging-related genes in *C. elegans***.** Relative mRNA expression levels of selected IIS-related and antioxidant/stress-response genes (*daf-2*, *ctl-2*, *hsp-16.2*, *daf-16*, *sod-3*, *akt-1*, and *skn-1*) were determined by qRT-PCR following EO-2141 treatment. Gene expression levels were normalized to those of the control group, which was set to 1. Data are presented as the mean ± SEM. * *p* < 0.05 and *** *p* < 0.001 versus the control group; ns indicates no significant difference (*p* > 0.05). Statistical significance was determined using an unpaired Student’s *t*-test.

The insulin/insulin-like signaling (IIS) pathway is a highly conserved regulatory network that plays a central role in controlling lifespan, stress resistance, and development in *C. elegans* [11]. To gain insight into the molecular basis underlying the antioxidant and longevity-promoting effects of EO-2141, the expression levels of selected IIS-related and antioxidant/stress-response genes were analyzed by qRT-PCR following treatment with 20 μg/mL EO-2141. As shown in Figure 6, gene expression levels were normalized to the control group. Treatment with EO-2141 significantly increased *daf-16* expression by 75.67%. The downstream stress-responsive gene *hsp-16.2* showed the strongest response, with a 4.4-fold increase in mRNA expression relative to the control group. In addition, *ctl-2* and *sod-3* expression levels were significantly upregulated by 60.33% and 20.33%, respectively. Conversely, *akt-1* expression was reduced by 28.33% (*p* < 0.001). No significant differences were observed in *daf-2* or *skn-1* expression. EO-2141 thus altered the expression of several antioxidant- and stress-response-related genes, including reduced *akt-1* expression, in *C. elegans*.

### 2.9. EO-2141 Promotes DAF-16 Nuclear Localization in C. elegans

**Figure 7:**
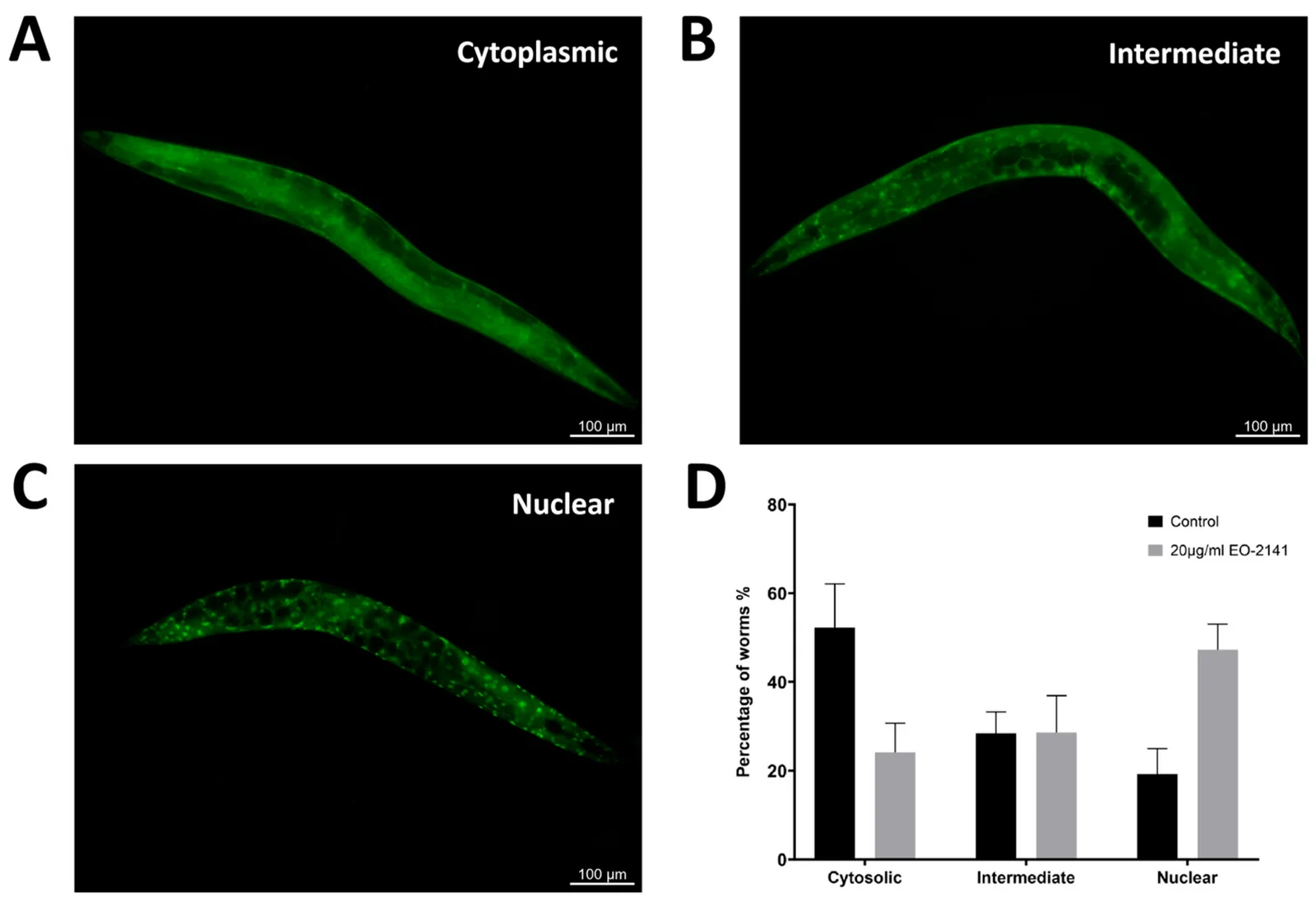
Effect of EO-2141 on DAF-16::GFP nuclear localization in *C. elegans*. Representative fluorescence images showing (**A**) predominantly cytoplasmic, (**B**) intermediate, and (**C**) predominantly nuclear localization of DAF-16::GFP. (**D**) Proportions of worms exhibiting cytoplasmic, intermediate, and nuclear DAF-16::GFP localization in the control and EO-2141-treated groups. Data are presented as mean ± SEM from three independent biological replicates, with approximately 30 worms examined per treatment group in each replicate. Statistical significance was determined using Pearson’s chi-square test. Scale bar = 100 μm.

DAF-16 is a key transcription factor involved in the regulation of stress responses and longevity in *C. elegans* [13]. To determine whether EO-2141 affected the subcellular localization of DAF-16, DAF-16::GFP localization was examined in the transgenic strain TJ356 following treatment with 20 μg/mL EO-2141. Representative fluorescence images showed a predominantly cytoplasmic distribution of DAF-16::GFP in control worms, whereas increased nuclear localization was observed following EO-2141 treatment (Figure 7A–C). Quantitative analysis showed that the overall distribution of DAF-16::GFP localization differed significantly between the control and EO-2141-treated groups (Pearson’s chi-square test, χ^2^(2) = 19.10, *p* = 0.000071; Figure 7D). The proportion of worms exhibiting nuclear DAF-16::GFP localization increased from 19.29% in the control group to 47.31% in the EO-2141-treated group, whereas the proportion exhibiting cytoplasmic localization decreased from 52.27% to 24.12%. The proportion showing intermediate localization remained similar (28.44% vs. 28.57%). These results indicate that EO-2141 promotes the redistribution of DAF-16 from the cytoplasm to the nucleus in *C. elegans*.

### 2.10. EO-2141 Does Not Significantly Extend Lifespan in daf-16(mgDf50) Mutant Worms

**Figure 8:**
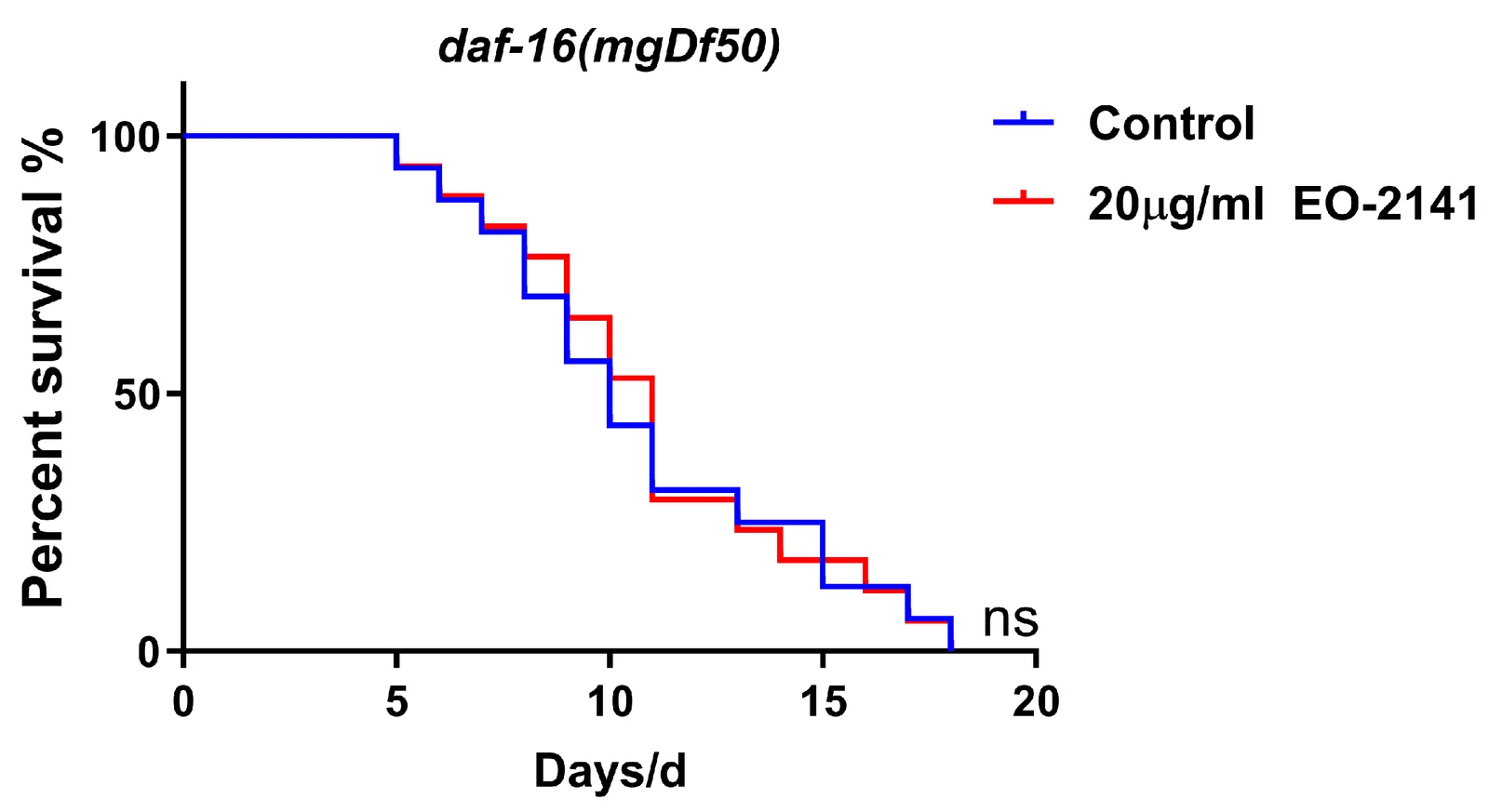
Effects of EO-2141 on the lifespan of *daf-16(mgDf50)* mutant worms (GR1307). Survival curves of *daf-16(mgDf50)* mutant worms treated with 20 μg/mL EO-2141. The control group consisted of worms maintained on standard NGM plates containing vehicle only (0.1% ethanol). Statistical significance was determined using the log-rank test. ns, not significant (*p* > 0.05).

To further assess the involvement of DAF-16 in EO-2141-mediated lifespan extension, lifespan assays were conducted using the loss-of-function *daf-16(mgDf50)* mutant strain GR1307. As shown in Figure 8 and Table 3, treatment with 20 μg/mL EO-2141 did not significantly affect either the mean lifespan or median lifespan of *daf-16(mgDf50)* mutant worms. The mean lifespan was 10.75 ± 0.45 days in the control group and 10.94 ± 0.55 days in the EO-2141-treated group. Similarly, the median lifespan increased only marginally from 10 to 11 days. Statistical analysis indicated that these differences were not significant (log-rank test, *p* > 0.05). No significant lifespan extension was detected in the *daf-16(mgDf50)* mutant background, supporting the involvement of DAF-16 in the longevity response to EO-2141.

## 3. Discussion

Previous studies on *M. sanguineum* have mainly focused on its phytochemical composition and in vitro biological activities, whereas its antioxidant and aging-related effects at the whole-organism level remain poorly understood. *C. elegans* has been widely used to evaluate the effects of natural products on lifespan and oxidative stress [22,23]. We therefore used *C. elegans* as an in vivo model to evaluate EO-2141 as a whole essential oil, with a focus on its antioxidant and lifespan-promoting effects.

In EO-2141, (E)-2-hexenal and n-hexadecanoic acid were the major components. (E)-2-Hexenal has previously been reported to possess antimicrobial and antifungal activities [24], whereas n-hexadecanoic acid (palmitic acid) has been shown to enhance stress resistance and extend lifespan in *C. elegans* [25]. Notably, the reported effects of palmitic acid were associated with DAF-16/FOXO and SKN-1/NRF2 signaling [25], which is of interest in light of the DAF-16-related effects observed for EO-2141 in the present study. However, because EO-2141 was evaluated as a whole essential oil, the observed biological effects cannot be attributed to any specific constituent.

In the lifespan assay, EO-2141 significantly extended the lifespan of wild-type worms, with mean lifespan increasing by 26.33% at 20 μg/mL. EO-2141 also reduced age-related lipofuscin accumulation and improved survival under oxidative and thermal stress. Lipofuscin-associated autofluorescence has been widely used as an aging-related indicator in *C. elegans* [18,19]. Similar improvements in lifespan and stress resistance have been reported for other essential oils in *C. elegans*. Patchouli essential oil extended lifespan and improved stress resistance, while ginseng volatile oil prolonged lifespan and increased survival following heat stress [26,27]. A range of plant-derived phytochemicals have also been reported to influence lifespan and stress-related phenotypes in *C. elegans* [28]. Increased stress resistance may therefore be an important feature accompanying the lifespan-promoting effects of some natural products.

Abnormal ROS accumulation is closely associated with oxidative stress and aging [20,29]. In this study, EO-2141 significantly reduced H_2_DCFH-DA-derived DCF fluorescence in the worms, which is consistent with reduced intracellular oxidative stress. Redox homeostasis is also regulated by endogenous antioxidant defense systems, in which SOD, CAT, and GSH-PX are important antioxidant enzymes [2,13]. EO-2141 increased SOD and GSH-PX activities, whereas CAT activity was not significantly changed, and MDA levels were significantly reduced. The differential responses of SOD, GSH-PX, and CAT suggest that EO-2141 does not uniformly affect all antioxidant enzymes. The changes in DCF fluorescence, antioxidant enzyme activities, and MDA were consistent with improved redox homeostasis following EO-2141 treatment. Interestingly, although EO-2141 showed relatively limited direct antioxidant activity in vitro, it markedly reduced DCF fluorescence and MDA levels and enhanced stress resistance in vivo. The DPPH and ABTS assays assess direct radical-scavenging activity in cell-free chemical systems, whereas the lower concentrations used in *C. elegans* evaluate biological responses in an intact organism and therefore are not directly comparable. The observed in vivo effects may therefore not be explained entirely by direct free-radical scavenging, and endogenous antioxidant and stress-response mechanisms may also be involved.

DAF-16 is a major regulator of lifespan and stress resistance in *C. elegans* and has been implicated in the lifespan-promoting effects of natural products [11,30,31]. For example, clove essential oil was reported to extend lifespan and promote DAF-16 nuclear translocation in *C. elegans* [14]. Given the effects of EO-2141 on lifespan, stress resistance, and redox homeostasis, we further examined whether DAF-16 was involved in the lifespan response to EO-2141. Targeted qRT-PCR analysis showed that EO-2141 significantly increased the expression of *daf-16*, *sod-3*, *ctl-2*, and *hsp-16.2*, while decreasing *akt-1* expression. In contrast, *daf-2* and *skn-1* expression did not change significantly.

However, increased *daf-16* transcript abundance alone does not demonstrate DAF-16 activation, which is more directly reflected by its subcellular localization. EO-2141 promoted DAF-16 nuclear localization, as indicated by increased nuclear and decreased cytoplasmic DAF-16::GFP localization in TJ356 worms. Moreover, although EO-2141 significantly extended lifespan in wild-type worms, no significant lifespan extension was detected in *daf-16(mgDf50)* mutants. Together, the transcriptional, DAF-16::GFP localization, and mutant lifespan data support the involvement of DAF-16 in the lifespan-promoting effect of EO-2141. These results, however, do not establish canonical IIS as the upstream pathway through which EO-2141 regulates DAF-16. Within the *C. elegans* insulin-like signaling pathway, AKT-1 negatively regulates DAF-16 [32], and the decrease in *akt-1* expression observed here is therefore consistent with altered IIS-related regulation. However, *daf-2* expression did not change significantly, and changes in transcript abundance alone do not reflect the actual activity of IIS components. Similarly, the unchanged *skn-1* transcript levels do not exclude the possible involvement of SKN-1-related regulation or other parallel longevity and stress-response pathways.

In conclusion, EO-2141 from *M. sanguineum* showed antioxidant and lifespan-promoting effects in *C. elegans* and improved resistance to oxidative and thermal stress. EO-2141 promoted DAF-16 nuclear localization, while no significant lifespan extension was detected in *daf-16(mgDf50)* mutants, supporting the involvement of DAF-16 in its longevity response.

## 4. Materials and Methods

### 4.1. Plant Material and Essential Oil Extraction

The leaves and stems of *Melastoma sanguineum* Sims were collected from a wild population in October 2021 in Jiuzhou Town, Lingshan County, Qinzhou City, Guangxi Province, China. The plant material was authenticated by Prof. Hong Zhao (College of Marine Science, Shandong University, Weihai, China). A voucher specimen (No. VK202141) was deposited in the Herbarium of the College of Marine Science, Shandong University, Weihai, China. Fresh leaves and stems (1000 g) were subjected to hydrodistillation using a Clevenger-type apparatus for 4 h. Hydrodistillation yielded a pale-yellow essential oil at 0.15 ± 0.01 mL/kg fresh weight. The resulting whole essential oil was designated EO-2141 and stored in sealed amber vials at 4 °C until further analysis.

### 4.2. Chemical Constituent Analysis

The chemical composition of EO-2141 was analyzed by GC-MS and GC-FID [33].

GC-MS analysis was carried out on an Agilent 7890–5975C gas chromatograph–mass spectrometer (Agilent Technologies, Santa Clara, CA, USA) equipped with an HP-5MS capillary column (30 m × 0.25 mm, 0.25 μm). The injector and interface temperatures were set at 260 °C and 280 °C, respectively. The oven temperature was programmed from 50 °C (held for 4 min) to 280 °C at 6 °C/min and then held for 3 min. Helium was used as the carrier gas at a flow rate of 1.10 mL/min. Mass spectra were acquired in EI mode at 70 eV over the *m*/*z* range of 25–500, with a scan rate of 4.0 scans/s; the quadrupole temperature was maintained at 150 °C. A 1% (*w*/*v*) solution of EO-2141 in n-hexane was injected (0.3 μL, splitless mode) [34].

GC-FID analysis was performed on a PerkinElmer Clarus 500 gas chromatograph (PerkinElmer, Shelton, CT, USA) equipped with an HP-5 capillary column (30 m × 0.25 mm, 0.25 μm film thickness). The injector and detector temperatures were set at 260 °C and 305 °C, respectively. The oven temperature program and carrier-gas conditions were the same as those used for GC-MS analysis [35].

Compounds were identified using Agilent MassHunter Qualitative Analysis 10.0 software by comparing mass spectra and retention indices with those reported in the NIST/EPA/NIH 2020 and Adams libraries. Retention indices were calculated using a homologous series of n-alkanes (C8–C30) analyzed under the same chromatographic conditions. Mass-spectral assignments with a retrieval matching probability (R.match) value > 700 were further evaluated by RI comparison, where available, with an acceptable RI deviation of ±10.

### 4.3. In Vitro Antioxidant Activity Assays of EO-2141 (DPPH, ABTS, and FRAP)

The in vitro antioxidant activity of EO-2141 was evaluated using DPPH radical-scavenging, ABTS radical-scavenging, and ferric reducing antioxidant power (FRAP) assays [16]. EO-2141 was dissolved in absolute ethanol to prepare the test solutions, while butylated hydroxytoluene (BHT) and Trolox were used as positive controls. Solvent controls containing the same volume of absolute ethanol but without EO-2141 were included in all in vitro antioxidant assays.

For the DPPH assay, a 0.17 mmol/L DPPH stock solution was prepared in ethanol. Briefly, 50 μL of EO-2141 solution at different concentrations was mixed with 200 μL of DPPH working solution in a 96-well microplate. After incubation at 25 °C in the dark for 30 min, the absorbance was measured at 516 nm using a microplate reader (BioTek Instruments, Minneapolis, MN, USA). The DPPH radical-scavenging activity was calculated according to the scavenging-rate equation, and the IC_50_ value was determined by nonlinear regression analysis [36].

For the ABTS assay, the ABTS radical cation (ABTS) working solution was generated by mixing ABTS solution with potassium persulfate and allowing the mixture to react in the dark for 12 h at room temperature. Subsequently, 50 μL of EO-2141 solution at various concentrations was mixed with 200 μL of ABTS working solution. After incubation in the dark at room temperature for 6 min, the absorbance was recorded at 734 nm. Radical-scavenging activity was calculated, and the IC_50_ value was obtained by nonlinear curve fitting using GraphPad Prism 9.5 software [37].

For the FRAP assay, a Trolox standard curve was established before analysis. Briefly, 50 μL of EO-2141 solution was mixed with 200 μL of freshly prepared FRAP working reagent and incubated at 37 °C in the dark for 40 min. Absorbance was then measured at 593 nm. Ferric reducing antioxidant power was calculated from the Trolox standard curve and expressed as μmol Trolox equivalents per gram of essential oil (μmol TE/g).

Three independent experiments were performed for each assay, with each sample measured in technical triplicate. Results are expressed as mean ± SD.

$$\mathrm{Scavenging}\mathrm{activity}\;(\%)=\left[1-\frac{\mathrm{As}-\mathrm{Ab}}{\mathrm{Ac}}\right]\times 100$$

where *As*, *Ab*, and *Ac* denote the absorbance of the sample, blank, and control, respectively.

### 4.4. Strains and Growth Conditions

*Escherichia coli* OP50 was cultured overnight in Luria–Bertani (LB) medium at 37 °C. Approximately 50 μL of the overnight OP50 culture was applied to the center of each NGM plate and allowed to form a bacterial lawn before use. The nematode strains used in this study included wild-type N2, GR1307 [*daf-16(mgDf50)*], and TJ356 [zIs356 (*daf-16p::daf-16a/b::GFP + rol-6(su1006*))], all of which were obtained from the Caenorhabditis Genetics Center (CGC, University of Minnesota, Minneapolis, MN, USA). Worms were maintained on NGM plates seeded with OP50 and cultured at 20 °C under standard laboratory conditions [38].

NGM plates were prepared using standard nematode growth medium containing 3 g NaCl, 2.5 g Bacto peptone, 25 mL of 1 M potassium phosphate buffer (pH 6.0), and 17 g agar per liter. After autoclaving, cholesterol (1 mL, 5 mg/mL), CaCl_2_ (1 mL, 1 M), and MgSO_4_ (1 mL, 1 M) were added aseptically [39].

Age-synchronized worms were obtained using the method described by Porta-de-la-Riva et al. [40]. Briefly, eggs were allowed to hatch in M9 buffer and develop to the L1 larval stage at 20 °C. The synchronized larvae were then transferred onto OP50-seeded NGM plates and cultured at 20 °C until they reached the L4 stage for subsequent experiments.

Based on preliminary exploratory concentration screening and previous studies of plant-derived essential oils in *C. elegans* [26,27], EO-2141 concentrations of 10 and 20 μg/mL were selected as the working concentrations for subsequent biological evaluation. EO-2141 was dissolved in absolute ethanol to prepare a 20 mg/mL stock solution, sterilized by membrane filtration (0.22 μm), and stored at 4 °C until use. A 10 mg/mL working solution was prepared by diluting the stock solution with an equal volume of absolute ethanol immediately before use.

Appropriate volumes of EO-2141 stock or working solution were added to autoclaved NGM medium at a ratio of 1:1000 (*v*/*v*) before plate preparation, resulting in final EO-2141 concentrations of 10 μg/mL and 20 μg/mL. The final concentration of ethanol in all treatment and control plates was maintained at 0.1% (*v*/*v*). Vehicle control plates contained the same concentration of ethanol (0.1%, *v*/*v*) but no EO-2141, thereby excluding potential effects caused by the solvent itself. Plates were allowed to dry at room temperature before use.

### 4.5. Lifespan Assay

Age-synchronized L4-stage N2 worms were transferred onto OP50-seeded NGM plates containing either EO-2141 (10 or 20 μg/mL), vitamin C (VC, 0.25 mg/mL; positive control), or vehicle alone (0.1% ethanol; negative control). To prevent progeny production, all plates contained 0.1 mg/mL 5-fluoro-2′-deoxyuridine (FUDR). In each independent experiment, three replicate plates were used for each treatment group, with approximately 30–40 worms per plate. Individual worms were used as the units for survival analysis.

The day on which worms were transferred to the treatment plates was designated as Day 0 of the lifespan assay. Worms were transferred to freshly prepared plates every 2–3 days to ensure an adequate food supply and prevent contamination. Survival was assessed every two days by recording the numbers of live and dead worms on each plate until all worms had died. Worms that crawled off the agar surface, died from accidental causes, or were otherwise lost during the experiment were censored from lifespan analysis. Worms were considered dead when they failed to respond to gentle stimulation of the head region with a platinum wire pick [41]. The lifespan assay was independently repeated three times.

To further assess the involvement of DAF-16 in the lifespan-extending effect of EO-2141, the same lifespan assay was performed using the *daf-16(mgDf50)* mutant strain GR1307 under identical experimental conditions.

### 4.6. Determination of Lipofuscin Accumulation in C. elegans

Age-synchronized L4-stage worms were transferred onto OP50-seeded NGM plates containing either EO-2141 (10 or 20 μg/mL) or vehicle control (0.1% ethanol) and cultured until the eight-day-old adult stage. Worms were then mounted on microscope slides coated with 2% agarose and immobilized with 10 mM levamisole hydrochloride.

Lipofuscin autofluorescence in the intestinal region was observed using an inverted Axio Scope A1 fluorescence microscope (Carl Zeiss AG, Jena, Germany) [18,19]. Images were captured under excitation at 340 nm and emission at 430 nm. Fluorescence intensity was quantified using ImageJ software (version 1.54d, NIH, Bethesda, MD, USA) and used as an indicator of age-related lipofuscin accumulation. The experiment was independently performed three times, with 10 worms analyzed per treatment group in each biological replicate.

### 4.7. Oxidative Stress Resistance Assay

Age-synchronized L4-stage worms were transferred onto OP50-seeded NGM plates containing EO-2141 (10 or 20 μg/mL), vitamin C (VC, 0.25 mg/mL; positive control), or vehicle control (0.1% ethanol) and cultured until the two-day-old adult stage.

Approximately 20 worms were transferred into each well of a 96-well plate containing 50 mM paraquat solution to induce acute oxidative stress [42,43]. Three replicate wells were prepared for each treatment group in each experiment. The time at which worms were transferred into the paraquat-containing wells was designated as 0 h. Worm survival was monitored every 2 h until all worms had died. Survival rates were calculated to evaluate the effect of EO-2141 on oxidative stress resistance. The experiment was independently performed three times.

### 4.8. Thermal Stress Resistance Assay

Age-synchronized L4-stage worms were transferred onto OP50-seeded NGM plates containing EO-2141 (10 or 20 μg/mL), vitamin C (VC, 0.25 mg/mL; positive control), or vehicle control (0.1% ethanol) and cultured until the two-day-old adult stage. In each independent experiment, three replicate fresh NGM plates were prepared for each treatment group, with approximately 20 worms transferred onto each plate.

The plates were incubated at 35 °C for 5 h to induce thermal stress and then returned to 20 °C for a 24 h recovery period. The start of incubation at 35 °C was designated as 0 h. After the 24 h recovery period, the numbers of surviving and dead worms were recorded, and survival rates were calculated to assess thermal stress resistance. Worms were considered dead when they failed to respond to gentle stimulation with a platinum wire pick. The experiment was independently repeated three times [44].

### 4.9. Determination of Antioxidant Enzyme Activities and MDA Content

Age-synchronized L4-stage worms were transferred onto OP50-seeded NGM plates containing EO-2141 (10 or 20 μg/mL), vitamin C (VC, 0.25 mg/mL; positive control), or vehicle control (0.1% ethanol) and cultured until the two-day-old adult stage. For each treatment group, approximately 1000 worms were collected into 1.5 mL microcentrifuge tubes using M9 buffer in each of three independent biological replicates and washed three times to remove residual bacteria.

After removal of the supernatant, worm pellets were resuspended in 500 μL of freshly prepared PBST (PBS containing 0.1% Tween-20) buffer and homogenized on ice using a handheld homogenizer (Kimble, Vineland, NJ, USA). The homogenates were centrifuged at 12,000 rpm for 1 min at 4 °C, and the resulting supernatants were collected and kept on ice for subsequent analyses.

The activities of superoxide dismutase (SOD), catalase (CAT), and glutathione peroxidase (GSH-PX), as well as malondialdehyde (MDA) content, were determined using commercially available assay kits (Nanjing Jiancheng Bioengineering Institute, Nanjing, China) according to the manufacturer’s instructions. Protein concentrations were measured using the bicinchoninic acid (BCA) protein assay [45], and enzyme activities were normalized to total protein content and expressed as units per milligram protein. Each biological sample was measured in technical triplicate.

### 4.10. Assessment of Intracellular Oxidative Status Using H_2_DCFH-DA

Age-synchronized L4-stage worms were transferred onto OP50-seeded NGM plates containing EO-2141 (10 or 20 μg/mL) or vehicle control (0.1% ethanol) and cultured until the two-day-old adult stage.

Worms were collected into 1.5 mL microcentrifuge tubes using M9 buffer and washed repeatedly to remove residual OP50 bacteria. After removal of the supernatant, worms were incubated with 2′,7′-dichlorodihydrofluorescein diacetate (H_2_DCFH-DA) at a final concentration of 25 μM for 30 min at 20 °C in the dark. After incubation, worms were washed several times with M9 buffer to remove excess probe.

In each independent experiment, 20 worms were randomly selected from each treatment group, mounted on 2% agarose pads, and immobilized with 10 mM levamisole hydrochloride. DCF fluorescence was detected at an excitation wavelength of 485 nm and an emission wavelength of 525 nm [46]. Fluorescence intensity was quantified individually for each worm using ImageJ software (NIH, Bethesda, MD, USA). The experiment was independently repeated three times.

### 4.11. Quantitative Real-Time PCR Analysis

Age-synchronized L4-stage worms were transferred onto OP50-seeded NGM plates containing EO-2141 (20 μg/mL) or vehicle control (0.1% ethanol) and cultured at 20 °C until the two-day-old adult stage. For each treatment group, three independent biological replicates were prepared, with each replicate consisting of a pooled sample of approximately 5000 age-synchronized worms. Worms were then collected using M9 buffer and transferred into 1.5 mL microcentrifuge tubes for RNA extraction.

Total RNA was extracted using the illustra™ RNAspin Mini RNA Isolation Kit (GE Healthcare, Buckinghamshire, UK) according to the manufacturer’s instructions. The concentration and purity of RNA samples were determined spectrophotometrically by measuring absorbance at 260 and 280 nm, and RNA integrity was further verified by agarose gel electrophoresis.

First-strand cDNA was synthesized using the ABScript II cDNA First-Strand Synthesis Kit (ABclonal, Wuhan, China). The 20 μL reaction mixture consisted of 2 μL total RNA, 2 μL random primer, 10 μL ABScript II Reaction Mix (2×), 2 μL ABScript II Enzyme Mix (10×), 1 μL dNTPs (10 mM), and nuclease-free water to volume. Reverse transcription was performed at 25 °C for 5 min, followed by 42 °C for 60 min, and 80 °C for 5 min.

Quantitative real-time PCR (qRT-PCR) was performed using a StepOnePlus™ Real-Time PCR System (Applied Biosystems, Foster City, CA, USA). Each 20 μL qRT-PCR reaction contained 0.4 μL cDNA template, 10 μL 2X Universal SYBR Green Fast qPCR Mix (ABclonal, Wuhan, China), 0.4 μL each of forward and reverse primers (10 μM), and nuclease-free water to volume. The amplification program consisted of an initial denaturation at 95 °C for 2 min, followed by 40 cycles of 95 °C for 5 s, 60 °C for 30 s, and 72 °C for 30 s.

The relative expression levels of target genes were normalized to *act-1* and calculated using the 2^−ΔΔCt^ method [47]. Primer sequences used for qRT-PCR analysis are listed in Table S2. Each biological sample was analyzed in technical triplicate by qRT-PCR.

### 4.12. DAF-16::GFP Nuclear Localization Assay

The transgenic strain TJ356 [zIs356 (*daf-16p::daf-16a/b::GFP* + rol-6(su1006))] was used to evaluate the subcellular localization of DAF-16. Age-synchronized L4-stage worms were transferred onto OP50-seeded NGM plates containing EO-2141 (20 μg/mL) or vehicle control (0.1% ethanol) and cultured at 20 °C for 48 h. Approximately 30 worms were randomly selected from each replicate, immobilized with 10 mM levamisole hydrochloride, and mounted on 2% agarose pads [48].

DAF-16::GFP localization was examined using an Axio Scope.A1 fluorescence microscope (Carl Zeiss AG, Jena, Germany) with excitation and emission wavelengths of 485 and 535 nm, respectively. Images were acquired using an Axiocam 503 color digital camera (Carl Zeiss, Jena, Germany) and ZEN Blue software (version 2.3 Lite). Based on the subcellular distribution of DAF-16::GFP, worms were classified into three categories: cytoplasmic, intermediate, or nuclear localization. The proportion of worms in each category was calculated for each treatment group. Three independent biological replicates were performed, with approximately 30 worms examined per treatment group in each replicate.

### 4.13. Statistical Analysis

Statistical analyses were performed using GraphPad Prism 9.5 (GraphPad Software, San Diego, CA, USA). All biological experiments were independently performed three times. For assays involving technical replicates, technical measurements were averaged within each biological replicate before statistical analysis. Data are presented as mean ± SEM unless otherwise stated. Comparisons between two groups were performed using unpaired Student’s *t*-test, while multiple-group comparisons were performed using one-way analysis of variance (ANOVA) followed by Dunnett’s post hoc test. DAF-16::GFP localization data were analyzed as categorical data using Pearson’s chi-square test to compare the distribution of worms among cytoplasmic, intermediate, and nuclear localization categories between treatment groups. Survival curves were analyzed using the Kaplan–Meier method and compared using the log-rank test. Differences were considered statistically significant at *p* < 0.05.

## Figures and Tables

**Table 1:** In vitro antioxidant activity of *M. sanguineum* essential oil (EO-2141) determined by DPPH, ABTS, and FRAP assays.

Samples	DPPH IC_50_ (mg/mL) ^a^	ABTS IC_50_ (mg/mL) ^a^	FRAP (μmol TE/g)
EO-2141	>10	3.72 ± 0.232	62.88 ± 2.75
BHT ^b^	0.043 ± 0.0012	0.015 ± 0.0003	—
Trolox ^b^	0.012 ± 0.0008	0.010 ± 0.0005	—

^a^: Half-maximal inhibitory concentration (IC_50_), defined as the concentration that reduces the measured response by 50%; ^b^: positive control used in this assay.

**Table 2:** Effects of EO-2141 on the lifespan of *C. elegans*.

Strain	Treatment	Mean Lifespan (Days)	Median Lifespan (Days)	Significance
Wild-type N2	Control	14.24 ± 0.39	16	—
Wild-type N2	10 μg/mL EO-2141	16.55 ± 0.32	16	*
Wild-type N2	20 μg/mL EO-2141	17.99 ± 0.31	18	***
Wild-type N2	0.25 mg/mL VC	18.43 ± 0.55	19	**

* *p* < 0.05; ** *p* < 0.01; *** *p* < 0.001.

**Table 3:** Effects of EO-2141 on the lifespan of *daf-16(mgDf50)* mutant nematodes.

Strain	Treatment	Mean Lifespan (Days)	Median Lifespan (Days)	Significance
GR1307	Control	10.75 ± 0.45	10	—
GR1307	20 μg/mL EO-2141	10.94 ± 0.55	11	ns

## Data Availability

The data presented in this study are available in the main text and the Supplementary Materials of this article.

## Supplementary Materials

The following supporting information can be downloaded at https://www.mdpi.com/article/10.3390/molecules31203585/s1, Table S1. Chemical composition of *Melastoma sanguineum* essential oil (EO-2141). Table S2. Primer sequences used for qRT-PCR analysis. Figure S1. Effect of EO-2141 on locomotor activity in *C. elegans*. (A) Body-bend frequency. (B) Head-thrash frequency. Data are presented as mean ± SEM from three independent experiments. Statistical significance was analyzed by one-way ANOVA. ns, not significant (*p* > 0.05) compared with the control group. Figure S2. Effect of EO-2141 on pharyngeal pumping rate of *C. elegans*. Data are presented as mean ± SEM from three independent experiments. Statistical significance was analyzed by one-way ANOVA. ns, not significant (*p* > 0.05) compared with the control group. Figure S3. Effect of EO-2141 on body size in *C. elegans*. (A) Representative images of worms from different treatment groups. (B) Quantitative analysis of body size. Data are presented as mean ± SEM from three independent experiments. Statistical significance was analyzed by one-way ANOVA. ns, not significant (*p* > 0.05) compared with the control group. Figure S4. Effect of EO-2141 on reproductive capacity in *C. elegans*. Total brood size was determined by counting the number of progeny produced by individual worms throughout the reproductive period. Data are presented as mean ± SEM from three independent experiments. Statistical significance was analyzed by one-way ANOVA. ns, not significant (*p* > 0.05) compared with the control group. Figure S5. Effect of EO-2141 on food clearance in *C. elegans*. Changes in the optical density (OD600) of OP50 bacterial suspensions were monitored over time to evaluate food consumption by worms. Data are presented as mean ± SEM from three independent experiments. No significant differences were observed between EO-2141-treated groups and the control group (*p* > 0.05). Figure S6. Total ion chromatogram (TIC) of EO-2141 obtained by GC-MS analysis. Numbers above selected peaks indicate retention times (min). Figure S7. Representative experimental EI-MS spectra and corresponding library spectra of selected constituents of EO-2141: (A) (E)-2-hexenal; (B) 1-hepten-3-ol; (C) n-hexadecanoic acid; (D) phytol; (E) mandenol; (F) ethyl linolenate; and (G) squalene. In each mirror plot, the red upward-facing spectrum represents the experimentally acquired EI-MS spectrum, whereas the blue downward-facing spectrum represents the corresponding library spectrum. MF, match factor; RMF, reverse match factor. Refs. [49,50,51,52,53,54,55] are cited in the Supplementary Materials.
