# Supplementary material for: Antioxidant Activity and DAF-16-Associated Lifespan-Promoting Effects of Essential Oil from Melastoma sanguineum Sims in Caenorhabditis elegans
Source: Molecules. 2026 Oct 9;31(20):3585. doi: 10.3390/molecules31203585 (PMC13707858; doi:10.3390/molecules31203585)
Supplement: Supplementary file 1 [file molecules-31-03585-s001.zip › molecules-4587995-supplementary.pdf]

Supplementary Data

Supplementary Results

1. Chemical Composition of Melastoma sanguineum Essential Oil (EO-2141)

The chemical composition of *Melastoma sanguineum* essential oil (EO-2141) was analyzed by GC-MS and GC-FID. A total of 91 compounds were identified (Table S1), including aldehydes, fatty acids, alkenes, and other volatile constituents. The identified compounds accounted for 98.99% of the total oil composition. Relative percentages of the individual components were calculated from GC-FID peak areas. The major constituents were *(E)*-2-hexenal (21.31%), *n*-hexadecanoic acid (16.40%), ethyl linolenate (3.61%), mandenol (3.56%), and squalene (3.19%).

Table S1. Chemical composition of *Melastoma sanguineum* essential oil (EO-2141)

No.	Compound name	%	RI	RI_{refer}	Identification method
1	2-Hexyn-1-ol	0.50	847	847	RRI, MS
2	(E)-2-Hexenal	21.31	—	854	MS
3	1-Hepten-3-ol	2.57	874	883	RRI, MS
4	Sorbaldehyde	0.36	913	911	RRI, MS
5	α-Pinene	0.33	934	937	RRI, MS
6	2-Hexen-4-olide	0.26	966	966	RRI, MS
7	Morillol	1.34	983	980	RRI, MS
8	Eucalyptol	0.44	1030	1032	RRI, MS
9	(E)-2-Octenal	0.23	1060	1060	RRI, MS
10	cis-Sabinene hydrate	0.30	1071	1070	RRI, MS
11	Linalool oxide	0.55	1074	1074	RRI, MS
12	p-Cymenene	0.52	1087	1090	RRI, MS
13	α-Pinene oxide	0.21	1094	1095	RRI, MS
14	Linalool	0.60	1098	1099	RRI, MS
15	Nonanal	0.55	1104	1104	RRI, MS
16	(E)-2-Nonenal	0.23	1161	1162	RRI, MS
17	α-Terpineol	0.32	1191	1189	RRI, MS
18	Methyl salicylate	1.50	1195	1192	RRI, MS
19	Fragranol	0.67	1216	1216	RRI, MS

20	p-Menth-1-en-9-al	0.56	1218	1225	RRI, MS
21	(E)-2-Decenal	0.55	1263	1263	RRI, MS
22	Longipinene	0.23	1350	1353	RRI, MS
23	Dehydro-ar-ionene	0.36	1353	1354	RRI, MS
24	Copaene	0.49	1375	1376	RRI, MS
25	β-Elemene	0.19	1391	1391	RRI, MS
26	Longifolene	1.64	1406	1406	RRI, MS
27	Caryophyllene	1.62	1421	1419	RRI, MS
28	2,2,3,3-Tetramethyl-1-indanone	0.35	1429	—	MS
29	Aromandendrene	0.21	1440	1440	RRI, MS
30	3-(4-Methoxyphenyl) cyclopentene	0.52	1448	1446	RRI, MS
31	Humulene	1.16	1454	1454	RRI, MS
32	Alloaromadendrene	0.21	1462	1461	RRI, MS
33	γ-Muurolene	0.53	1479	1477	RRI, MS
34	β-Guaiene	0.18	1483	1481	RRI, MS
35	β-Selinene	0.43	1487	1486	RRI, MS
36	Viridiflorene	0.65	1496	1493	RRI, MS
37	α-Muurolene	0.39	1502	1499	RRI, MS
38	α-Farnesene	0.43	1508	1508	RRI, MS
39	Cubebol	1.02	1515	1515	RRI, MS
40	Zonarene	0.94	1527	1527	RRI, MS
41	α-Cadinene	0.21	1538	1538	RRI, MS
42	α-Calacorene	0.19	1547	1542	RRI, MS
43	Nerolidol	0.26	1562	1563	RRI, MS
44	trans-Nerolidol	0.55	1566	1564	RRI, MS
45	Palustrol	0.27	1571	1568	RRI, MS
46	(3E,7E)-4,8,12-Trimethyltrideca-1,3,7,11-tetraene	0.31	1580	1577	RRI, MS
47	Epiglobulol	0.46	1587	1585	RRI, MS

48	Guaiol	0.37	1597	1596	RRI, MS
49	β-Biotol	0.21	1613	1612	RRI, MS
50	epi-Cedrol	0.65	1617	1618	RRI, MS
51	trans-Isolongifolanone	0.18	1627	1626	RRI, MS
52	2-Selin-6-en-4α-ol	0.20	1636	1636	RRI, MS
53	T-Cadinol	0.67	1646	1642	RRI, MS
54	α-Cadinol	0.43	1660	1653	RRI, MS
55	Costal	0.48	1677	1691	RRI, MS
56	Heptadecane	1.58	1701	1700	RRI, MS
57	14-hydroxy-α-Humulene	0.19	1715	1714	RRI, MS
58	cis, trans-Farnesal	0.33	1728	1727	RRI, MS
59	Tetradecanoic acid	1.20	1772	1768	RRI, MS
60	Hexadecanal	0.21	1815	1817	RRI, MS
61	Neophytadiene	0.19	1837	1838	RRI, MS
62	Hexahydrofarnesyl acetone	0.79	1845	1844	RRI, MS
63	Pentadecanoic acid	0.46	1868	1867	RRI, MS
64	Carbonic acid, ethyl tridecyl ester	0.19	1871	—	MS
65	Nonadecane	0.64	1897	1900	RRI, MS
66	Farnesyl acetone	0.32	1919	1918	RRI, MS
67	Isophytol	1.39	1948	1948	RRI, MS
68	n-Hexadecanoic acid	16.40	1969	1968	RRI, MS
69	Eicosane	0.27	2001	2000	RRI, MS
70	Isopropyl palmitate	0.24	2019	2024	RRI, MS
71	Geranyl linalool	0.21	2031	2034	RRI, MS
72	Heptadecanoic acid	0.29	2070	2071	RRI, MS
73	1-Octadecanol	0.51	2086	2082	RRI, MS
74	Heneicosane	0.37	2100	2100	RRI, MS
75	Phytol	2.88	2117	2114	RRI, MS
76	Linoleic acid	1.71	2134	2133	RRI, MS

77	Mandenol	3.56	2155	2160	RRI, MS
78	Ethyl linolenate	3.61	2161	2169	RRI, MS
79	Octadecanoic acid	0.45	2174	2172	RRI, MS
80	Docosane	0.37	2200	2200	RRI, MS
81	(Z)-9-Tricosene	0.30	2273	2275	RRI, MS
82	Tricosane	1.27	2300	2300	RRI, MS
83	cis-13-Eicosenoic acid	0.30	2366	2366	RRI, MS
84	Tetracosane	0.84	2400	2400	RRI, MS
85	Pentacosane	0.74	2499	2500	RRI, MS
86	Hexacosane	0.34	2598	2600	RRI, MS
87	Heptacosane	0.55	2699	2700	RRI, MS
88	Octacosane	0.70	2799	2800	RRI, MS
89	Squalene	3.19	2836	2827	RRI, MS
90	Nonacosane	0.86	2900	2900	RRI, MS
91	Hentriacontane	0.65	—	3100	MS

Notes: Relative contents (%) were determined by GC-FID peak-area normalization. RI denotes the retention index experimentally determined on an HP-5MS column, whereas RI*refer* denotes the retention index referenced from the NIST/EPA/NIH 2020 Mass Spectral Database. —, not determined. Compound identification was based on comparison of retention indices and mass spectra with the NIST/EPA/NIH 2020 Mass Spectral Library and published literature data. "RRI, MS" indicates identification supported by both RI agreement and mass-spectral library matching, whereas "MS" indicates identification based on mass-spectral library matching without experimental RI confirmation.

Table S2. Primer sequences used for qRT-PCR analysis.

Gene	Forward Primer	Reverse Primer	Source
**act-1*	CCCCACTCAATCCAAAGGCT	GTACGTCCGGAAGCGTAGAG	Ref. [49]
sod-3	GGCTAAGGATGGTGGAGAAC	ACAGGTGGCGATCTTCAAG	Ref. [50]
ctl-2	GAGAATGTGCCAGAACTTTGC	CTTGACACGAGCTCCAAAATC	Ref. [51]
skn-1	GACGTCAATTTATGGAGTGTCG	GAAGATGTTTTGTCGTGATCCG	Ref. [50]
daf-2	GGATAAAGGCGAATCAAAGTGTC	CGATACACTTTCCCTTGTGATAGAC	Ref. [51]
daf-16	TCAAGCCAATGCCACTACC	TGGAAGAGCCGATGAAGAAG	Ref. [50]
hsp-16.2	TATGGCTCTGATGGAACG	GATTGATAGCGTACGACC	Ref. [52]

akt-1	TCACCGATGCGATATTGTCT	AACTCCCCACCAATCAACAC	Ref. [53]

Note: **act-1* was used as the internal reference gene. Primer sequences were adopted from previously published *C. elegans* qRT-PCR studies listed in the Source column. Each sample was analyzed in triplicate.

2. Effect of EO-2141 on Locomotor Activity in C. elegans

Locomotor activity is a commonly used physiological indicator of aging in *C. elegans*. During the aging process, worms gradually exhibit declines in coordinated movement, which are reflected by reduced frequencies of body bends and head thrashes. To evaluate whether EO-2141 affected locomotor performance, age-synchronized worms were exposed to EO-2141, and the numbers of body bends and head thrashes were recorded on untreated NGM plates. As shown in Figure S1, treatment with EO-2141 at 10 or 20 μg/mL did not significantly affect either body-bend frequency or head-thrash frequency compared with the control group ($p > 0.05$). These results indicate that EO-2141 has no detectable effect on locomotor activity under the experimental conditions used.

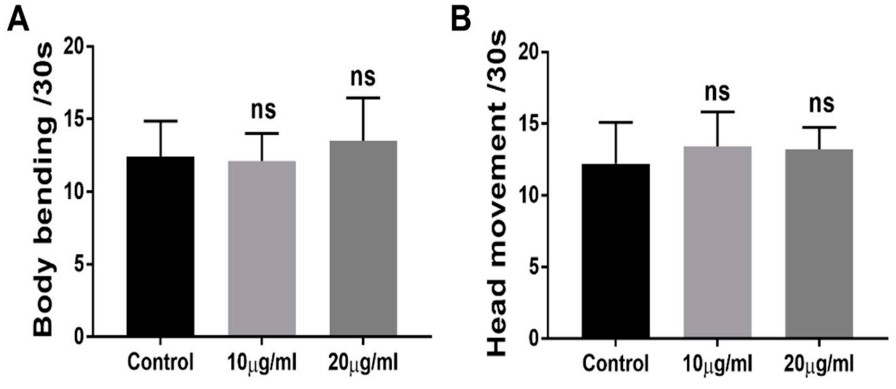

Figure S1. Effect of EO-2141 on locomotor activity in *C. elegans*. (A) Body-bend frequency. (B) Head-thrash frequency. Data are presented as mean ± SEM from three independent experiments. Statistical significance was analyzed by one-way ANOVA. ns, not significant ($p > 0.05$) compared with the control group.

3. Effect of EO-2141 on Pharyngeal Pumping Rate of C. elegans

The pharyngeal pumping rate is widely used as an indicator of feeding activity and age-related functional decline in *C. elegans*. As worms age, the pumping rate gradually decreases owing to deterioration of the pharyngeal musculature. To determine whether EO-2141 affects feeding behavior or muscle function, the pharyngeal pumping rate was measured in worms treated with EO-2141. As shown in Figure S2, treatment with EO-2141 at concentrations of 10 or 20 μg/mL did not significantly alter the pharyngeal pumping rate compared with the control group ($p > 0.05$). These results indicate that EO-2141 has no detectable effect on feeding activity or pharyngeal function under the experimental conditions used.

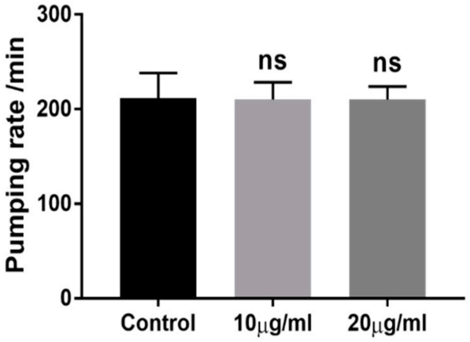

Figure S2. Effect of EO-2141 on pharyngeal pumping rate of *C. elegans*. Data are presented as mean ± SEM from three independent experiments. Statistical significance was analyzed by one-way ANOVA. ns, not significant ($p > 0.05$) compared with the control group.

4. Effect of EO-2141 on Body Size in C. elegans

External environmental stimuli may affect the growth and development of *C. elegans*, resulting in phenotypic differences in body size and body shape; certain pharmacological interventions can also disrupt their normal developmental progression. To determine whether the longevity effect of EO-2141 was accompanied by altered growth and development, worms at the two-day adult stage were randomly selected, imaged under a fluorescence microscope, and analyzed for body size (Figure S3A). Worms treated with 10 or 20 μg/mL EO-2141 showed no significant differences in body size relative to the control group (Figure S3B, $p > 0.05$). These findings indicate that EO-2141, at the tested concentrations, does not interfere with normal growth and development in *C. elegans*.

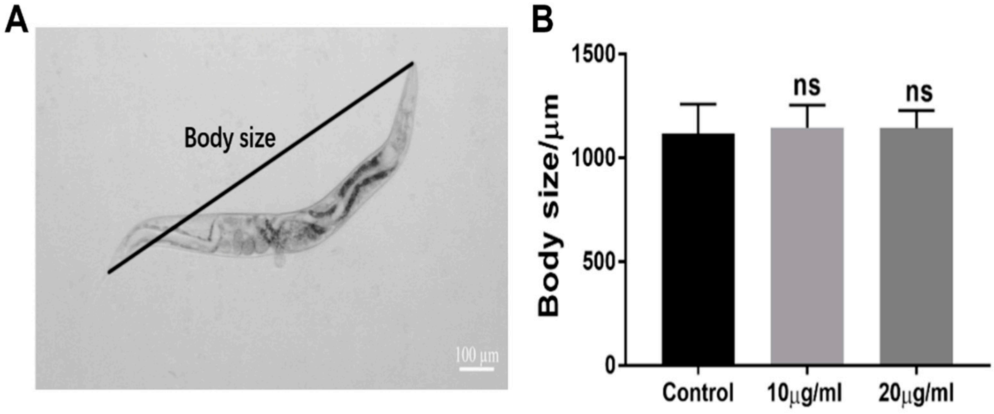

Figure S3. Effect of EO-2141 on body size in *C. elegans*. (A) Representative images of worms from different treatment groups. (B) Quantitative analysis of body size. Data are presented as mean ± SEM from three independent experiments. Statistical significance was analyzed by one-way ANOVA. ns, not significant ($p > 0.05$) compared with the control group.

5. Effect of EO-2141 on Reproductive Capacity in C. elegans

Reproductive capacity is an important indicator of organismal fitness and is commonly used to evaluate potential trade-offs associated with lifespan extension in *C. elegans*. To determine whether EO-2141 affects reproductive performance, the daily brood size and total progeny production of individual worms were recorded throughout the reproductive period. As shown in Figure S4, the average total brood sizes of worms treated with 10 and 20 μg/mL EO-2141 were 244 and 237 progeny per worm, respectively, compared with 237 progeny per worm in the control group. Statistical analysis revealed no significant differences among the groups ($p > 0.05$). These results indicate that EO-2141 does not significantly affect reproductive capacity in *C. elegans* under the experimental conditions used. Furthermore, EO-2141-induced lifespan extension was not accompanied by reduced reproductive output, suggesting that longevity promotion occurred without an apparent reproduction-related fitness cost.

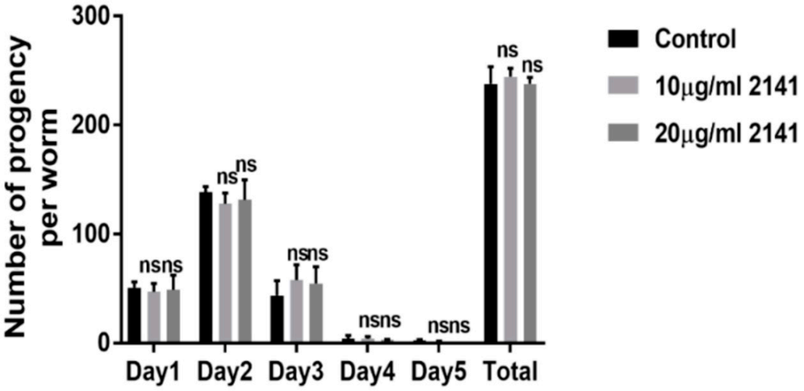

Figure S4. Effect of EO-2141 on reproductive capacity in *C. elegans*. Total brood size was determined by counting the number of progeny produced by individual worms throughout the reproductive period. Data are presented as mean ± SEM from three independent experiments. Statistical significance was analyzed by one-way ANOVA. ns, not significant ($p > 0.05$) compared with the control group.

6. Effect of EO-2141 on Food Clearance in C. elegans

Dietary restriction has been widely reported to extend lifespan and delay aging in *C. elegans*. Because Escherichia coli OP50 serves as the primary food source for laboratory-cultured worms, changes in bacterial consumption may influence lifespan outcomes. To determine whether the lifespan-extending effect of EO-2141 was associated with altered food intake, the food clearance assay was performed by monitoring changes in the optical density (OD600) of OP50 bacterial suspensions over time. As shown in Figure S5, the OD600 values of bacterial cultures decreased gradually in all groups as food was consumed by the worms. However, no significant differences in the rate of OD600 decline were observed between the EO-2141-treated groups (10 and 20 μg/mL) and the control group throughout the experimental period ($p > 0.05$). These results indicate that EO-2141 did not significantly affect food consumption or feeding behavior in *C. elegans*. Therefore, the lifespan extension induced by EO-2141 is unlikely to result from dietary restriction or reduced energy intake.

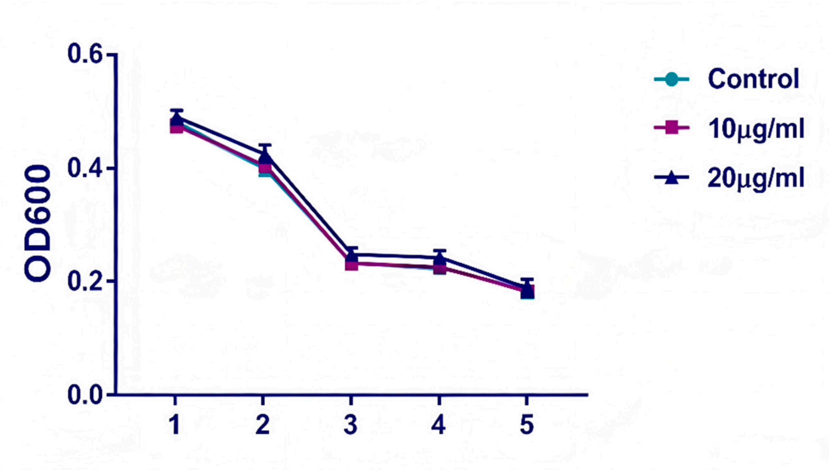

Figure S5. Effect of EO-2141 on food clearance in *C. elegans*. Changes in the optical density (OD600) of OP50 bacterial suspensions were monitored over time to evaluate food consumption by worms. Data are presented as mean ± SEM from three independent experiments. No significant differences were observed between EO-2141-treated groups and the control group ($p > 0.05$).

7. GC-MS Chromatogram and Representative Mass Spectra of EO-2141

The total ion chromatogram (TIC) of EO-2141 is shown in Figure S6. Representative experimental EI-MS spectra and the corresponding library spectra of seven relatively abundant constituents are shown in Figure S7. These compounds include (E)-2-hexenal, 1-hepten-3-ol, n-hexadecanoic acid, phytol, mandenol, ethyl linolenate, and squalene.

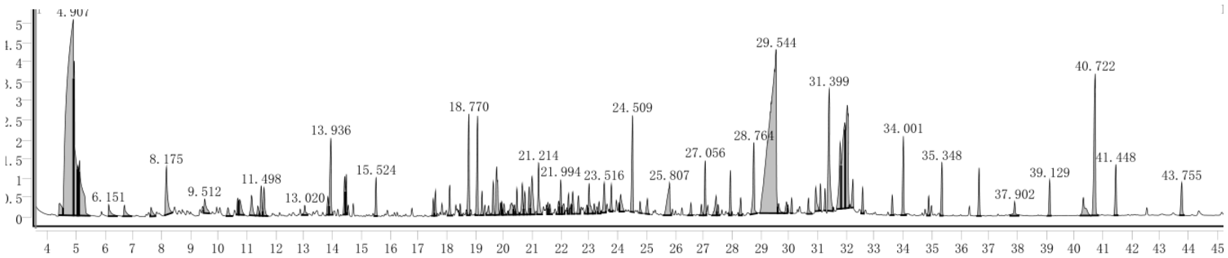

Figure S6. Total ion chromatogram (TIC) of EO-2141 obtained by GC-MS analysis. Numbers above selected peaks indicate retention times (min).

(A) No. 2, (E)-2-Hexenal (MF = 945; RMF = 954)

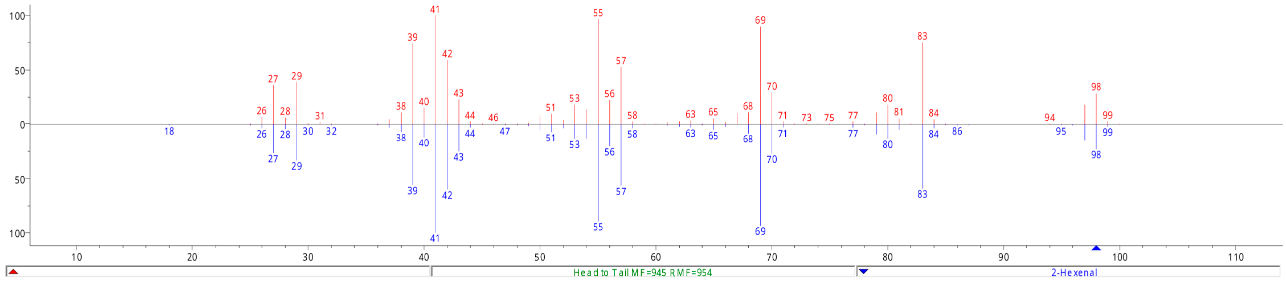

(B) No. 3, 1-Hepten-3-ol (MF = 962; RMF = 962)

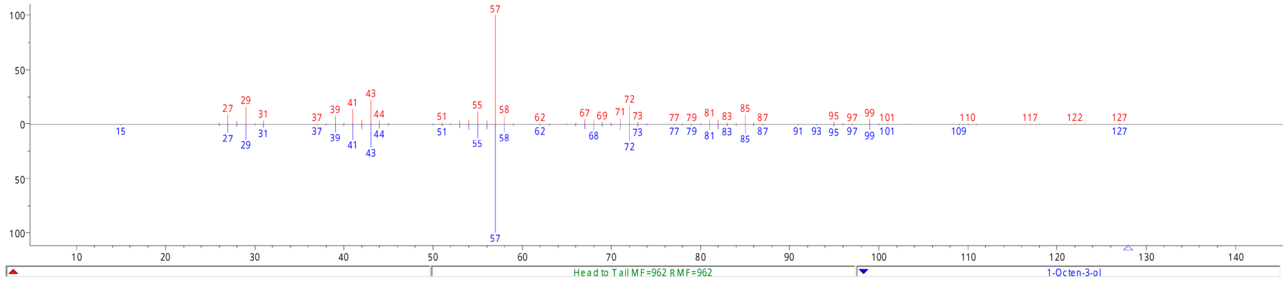

(C) No. 68, n-Hexadecanoic acid (MF = 947; RMF = 954)

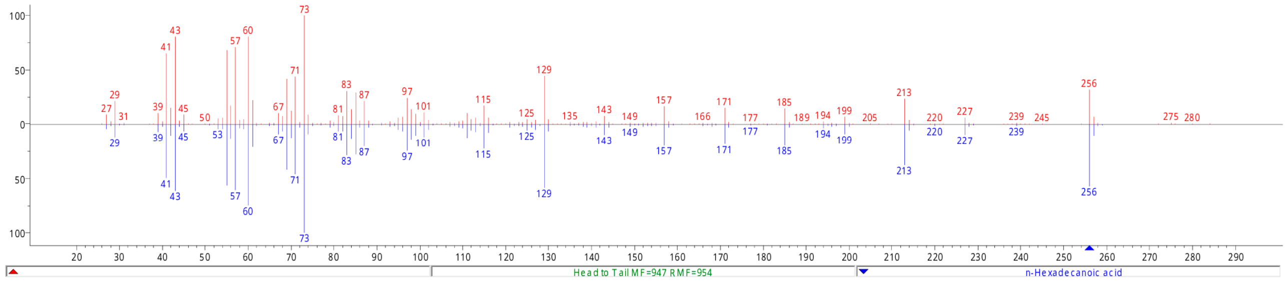

(D) No. 75, Phytol (MF = 942; RMF = 955)

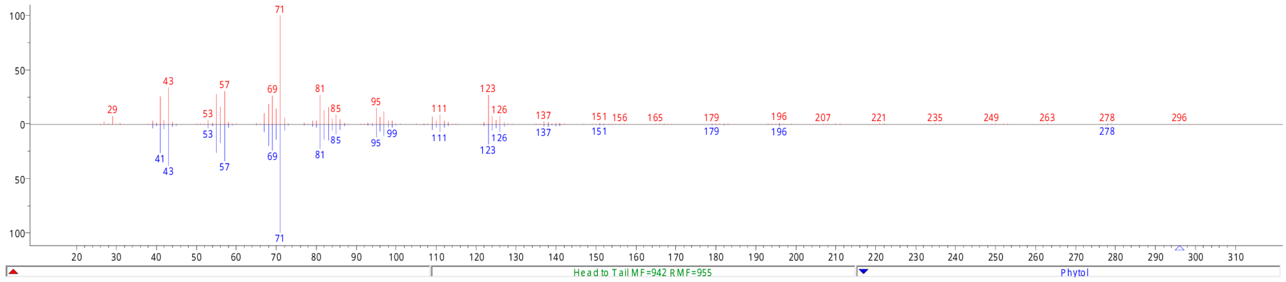

(E) No. 77, Mandenol (MF = 754; RMF = 800)

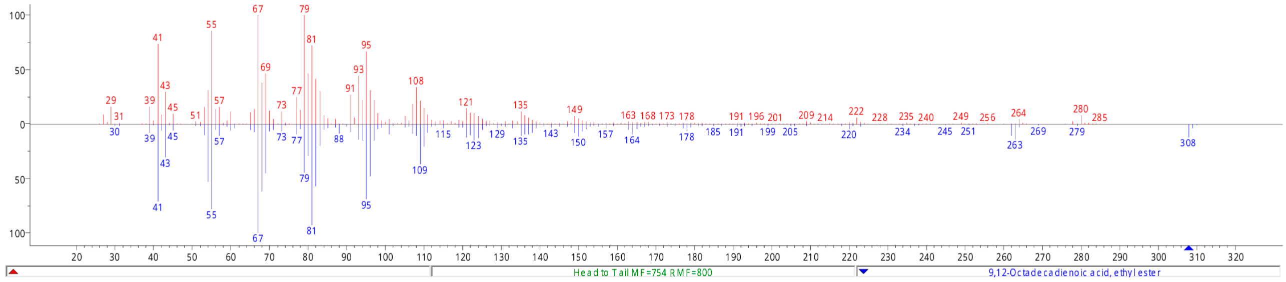

(F) No. 78, Ethyl linolenate (MF = 727; RMF = 732)

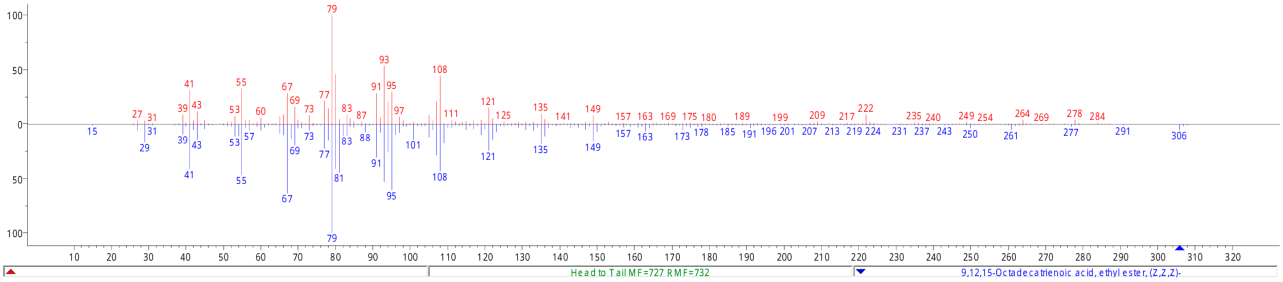

(G) No. 89, Squalene (MF = 956; RMF = 956)

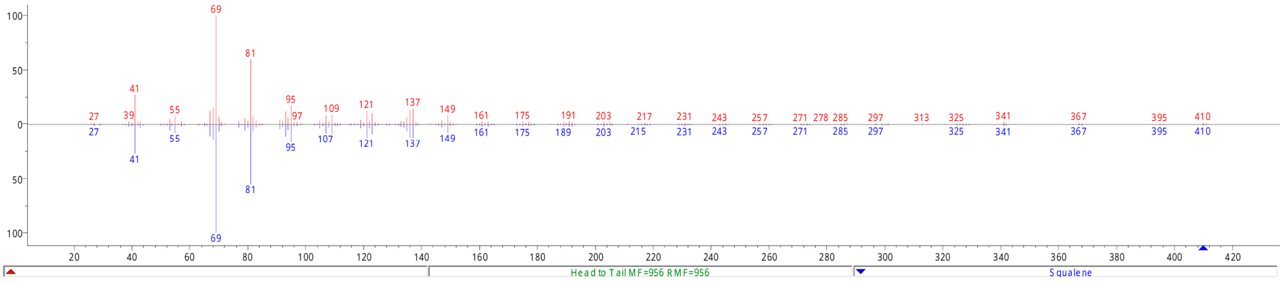

Figure S7. Representative experimental EI-MS spectra and corresponding library spectra of selected constituents of EO-2141: (A) (E)-2-hexenal; (B) 1-hepten-3-ol; (C) n-hexadecanoic acid; (D) phytol; (E) mandenol; (F) ethyl linolenate; and (G) squalene. In each mirror plot, the red upward-facing spectrum represents the experimentally acquired EI-MS spectrum, whereas the blue downward-facing spectrum represents the corresponding library spectrum. MF, match factor; RMF, reverse match factor.

Supplementary Methods

1. Locomotor Activity Assay

Age-synchronized L4-stage worms were transferred onto OP50-seeded NGM plates containing EO-2141 (10 or 20 μg/mL) or vehicle control (0.1% ethanol) and cultured until the two-day-old adult stage. For each treatment group, 10 worms were randomly selected and transferred onto fresh NGM plates without bacterial lawns. After a 1 min acclimation period, the numbers of body bends and head thrashes were counted for 30 s [54]. A body bend was defined as a change in the direction of the worm body relative to the longitudinal axis, whereas a head thrash was defined as a lateral movement of the head away from the direction of forward locomotion. The experiment was independently repeated three times.

2. Pharyngeal Pumping Assay

Age-synchronized L4-stage worms were transferred onto OP50-seeded NGM plates containing EO-2141 (10 or 20 μg/mL) or vehicle control (0.1% ethanol) and cultured until the two-day-old adult stage. Individual worms were transferred onto fresh OP50-seeded NGM plates and allowed to acclimate for 30 min. Pharyngeal pumping activity was then observed under a stereomicroscope (×400 magnification), and the number of pharyngeal contractions

was counted continuously for 1 min [55]. The experiment was independently repeated three times, with 10 worms analyzed per treatment group in each biological replicate.

3. Body size Measurement

Age-synchronized L4-stage worms were transferred onto OP50-seeded NGM plates containing EO-2141 (10 or 20 μg/mL) or vehicle control (0.1% ethanol) and cultured until the two-day-old adult stage. Approximately 20 worms from each group were mounted on agarose pads and immobilized with 10 mM levamisole hydrochloride. Images were captured using an inverted fluorescence microscope. Body size was measured using ImageJ software from the anterior tip of the mouth to the tail tip along the body midline. The experiment was independently repeated three times.

4. Reproductive Capacity Assay

Age-synchronized L1 larvae were transferred onto OP50-seeded NGM plates containing EO-2141 (10 or 20 μg/mL) or vehicle control (0.1% ethanol). For each treatment group, five worms were placed on each plate, with three replicate plates per group. Worms were transferred to fresh plates every 12 h until egg laying ceased. The number of eggs laid on each plate was counted every 12 h, and the data were pooled every 24 h to determine the daily brood size. The average daily brood size and total brood size per worm were then calculated. The experiment was independently repeated three times.

5. Food Clearance Assay

Food clearance was assessed using a liquid culture system in 96-well plates. Age-synchronized L4-stage worms were suspended in S medium at a density of approximately 15–20 worms per 10 μL. The OP50 bacterial suspension was adjusted to an OD600 of 0.50–0.60. Treatment groups included vehicle control (0.1% ethanol), EO-2141 (10 μg/mL), and EO-2141 (20 μg/mL). Each group contained three replicate wells, and the experiment was independently repeated three times. The final volume in each well was 200 μL and contained OP50 suspension, S medium, FUDR, and the corresponding treatment solution. Plates were sealed and incubated at 20 °C with shaking. Bacterial density was monitored by measuring OD600 immediately after plate preparation (Day 0) and once daily for five consecutive days using a microplate reader.

References

49. Li, Y.X.; Wang, N.N.; Zhou, Y.X.; Lin, C.G.; Wu, J.S.; Chen, X.Q.; Chen, G.J.; Du, Z.J. *Planococcus maritimus* ML1206 isolated from wild oysters enhances the survival of *Caenorhabditis elegans* against *Vibrio anguillarum*. *Mar. Drugs.* **2021**, *19*, 150.

50. Wang, J.; Deng, N.; Wang, H.; Li, T.; Chen, L.; Zheng, B.; Liu, R.H. Effects of orange extracts on longevity, healthspan, and stress resistance in *Caenorhabditis elegans*. *Molecules* **2020**, *25*, 351.

51. Lin, C.; Zhang, X.; Su, Z.; Xiao, J.; Lv, M.; Cao, Y.; Chen, Y. Carnosol improved lifespan and healthspan by promoting antioxidant capacity in *Caenorhabditis elegans*. *Oxid. Med. Cell. Longev.* **2019**, *2019*, 5958043.

52. Qi, Z.; Ji, H.; Le, M.; et al. Sulforaphane promotes *C. elegans* longevity and healthspan via DAF-16/DAF-2 insulin/IGF-1 signaling. *Aging* **2021**, 13, 1649.

53. Li, J.; Ebata, A.; Dong, Y.; et al. *Caenorhabditis elegans* HCF-1 functions in longevity maintenance as a DAF-16 regulator. *PLoS Biol.* **2008**, 6, e233.

54. Herndon, L.A.; Schmeissner, P.J.; Dudaronek, J.M.; Brown, P.A.; Listner, K.M.; Sakano, Y.; Paupard,

M.C.; Hall, D.H.; Driscoll, M. Stochastic and genetic factors influence tissue-specific decline in ageing *C. elegans*. *Nature* **2002**, 419, 808-814.

55. Huang, C.; Xiong, C.; Kornfeld, K. Measurements of age-related changes of physiological processes that predict lifespan of *Caenorhabditis elegans*. *Proc. Natl. Acad. Sci. U.S.A.* **2004**, 101, 8084-8089.
